# Porous Microreactor Chip for Photocatalytic Seawater Splitting over 300 Hours at Atmospheric Pressure

**DOI:** 10.1007/s40820-025-01703-6

**Published:** 2025-03-17

**Authors:** Desheng Zhu, Zhipeng Dong, Chengmei Zhong, Junhong Zhang, Qi Chen, Ni Yin, Wencheng Jia, Xiong Zheng, Fengzai Lv, Zhong Chen, Zhenchao Dong, Wencai Huang

**Affiliations:** 1 Department of Electronic Engineering, School of Electronic Science and Technology, Xiamen University, Xiamen, 361005 People’s Republic of China; 2 Fuzhou Fuzhi Photocatalysis Research Center, Fuzhou, 350007 People’s Republic of China; 3 i-Lab, CAS Key Laboratory of Nanophotonic Materials and Devices, Suzhou Institute of Nano-Tech and Nano-Bionics, Chinese Academy of Sciences, Suzhou, 215123 People’s Republic of China; 4 Hefei National Research Center for Physical Sciences at the Microscale and CAS Center for Excellence in Quantum Information and Quantum Physics, University of Science and Technology of China, Hefei, 230026 People’s Republic of China; 5 State Key Laboratory of Physical Chemistry of Solid Surfaces, Xiamen University, Xiamen, 361005 People’s Republic of China

**Keywords:** Solar energy, Hydrogen energy, Photocatalytic seawater splitting, Silver phosphate/Cadmium sulfide heterojunction

## Abstract

Photocatalytic seawater splitting is an attractive way for producing green hydrogen. Significant progresses have been made recently in catalytic efficiencies, but the activity of catalysts can only maintain stable for about 10 h. Here, we develop a vacancy-engineered Ag_3_PO_4_/CdS porous microreactor chip photocatalyst, operating in seawater with a performance stability exceeding 300 h. This is achieved by the establishment of both catalytic selectivity for impurity ions and tailored interactions between vacancies and sulfur species. Efficient transport of carriers with strong redox ability is ensured by forming a heterojunction within a space charge region, where the visualization of potential distribution confirms the key design concept of our chip. Moreover, the separation of oxidation and reduction reactions in space inhibits the reverse recombination, making the chip capable of working at atmospheric pressure. Consequently, in the presence of Pt co-catalysts, a high solar-to-hydrogen efficiency of 0.81% can be achieved in the whole durability test. When using a fully solar-driven 256 cm^2^ hydrogen production prototype, a H_2_ evolution rate of 68.01 mmol h^−1^ m^−2^ can be achieved under outdoor insolation. Our findings provide a novel approach to achieve high selectivity, and demonstrate an efficient and scalable prototype suitable for practical solar H_2_ production.

**Supplementary Information:**

The online version contains supplementary material available at 10.1007/s40820-025-01703-6.

## Highlights

- A film-type Ag_3_PO_4_/CdS photocatalyst in a porous substrate configuration is designed and prepared to avoid photocorrosion and ensure outstanding firmness.
- Both the S and Ag vacancy-rich surface and the heterojunction within the space charge region are found to be the key for high solar-to-hydrogen efficiency and excellent stability in seawater over 300 h.
- The first fully solar-driven hydrogen production prototype is constructed to work outdoors, exhibiting a high H_2_ evolution rate of 68.01 mmol h^−1^ m^−2^.

**Supplementary Information:**

The online version contains supplementary material available at 10.1007/s40820-025-01703-6.

## Graphical abstract

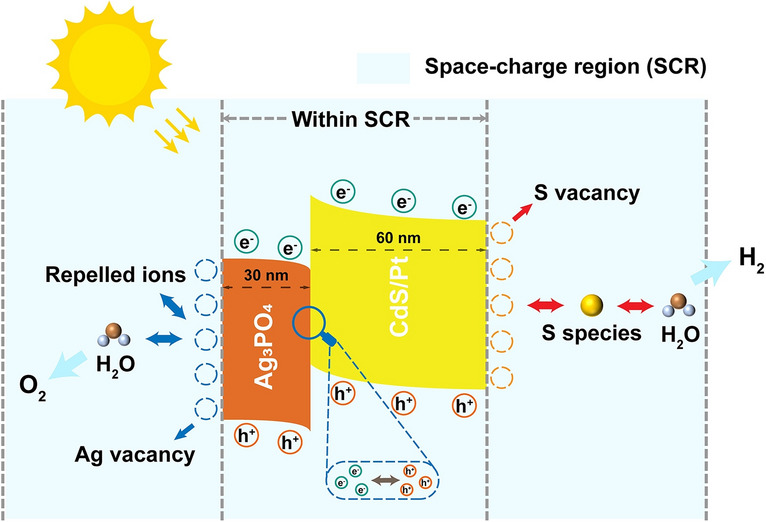

## Introduction

Using inexhaustible and widely available renewable energy to provide long-term clean fuel for mankind is an invaluable quest. The Honda-Fujishima effect [1], proposed in early 1970s for water splitting via the photoelectrochemical (PEC) approach [2–6], offers a promising solution for the above-mentioned pursuit. Aiming to readily convert solar energy and water into chemical energy, extensive researches have been carried out, with a focus on bias-free unassisted photocatalytic overall water splitting (OWS) [7–14]. Such a technology can produce H_2_ and O_2_ at a molar ratio of 2:1 in one-step solar-to-chemical energy conversion. Photocatalytic OWS has achieved an inspiring solar-to-hydrogen (STH) efficiency exceeding 1% [15], with the major challenges being the rapid recombination of photogenerated carriers during transport and the conflict between the broad visible-light absorption and strong redox ability [16, 17]. Besides, the feedstock for most photocatalytic OWS systems is pure water [18, 19]. Abundant seawater is a promising substitute for increasingly scarce freshwater resources, but the bottleneck issue is to suppress or even avoid the sharp deactivation of photocatalysts induced by the high salinity of seawater.

Limited by the disorder kinetic behavior of photogenerated carriers, the stability of early reported half-reaction systems in seawater was mostly less than or equal to 6 h [18]. Coupling two individual photocatalysts to form a Z-scheme heterojunction [20–22] has recently been proposed to generate both H_2_ and O_2_. The Z-scheme configuration is superior in addressing the low photocatalytic activity, but has so far not been possible to achieve a cycle that lasts for tens of hours [23] due to the undesirable competing reactions of Cl species. Very recently, Mi et al. reported a series of work with InGaN/GaN nanowires (NWs) as the major photocatalysts [24, 25]. When assisted with a Rh/Cr_2_O_3_/Co_3_O_4_ cocatalyst, the STH efficiency was as high as 6.6%, but only tested for 10 h artificial seawater splitting. The efficiency improvement is probably due to both the high reaction temperature at 70 °C and the good lattice-matched structure between InGaN and GaN fabricated by molecular-beam-epitaxy technology. Nevertheless, an overlooked fact is that the durability of catalysts in seawater differs significantly from that in pure water, which is believed to associate with interface reaction processes [26]. The lack of catalytic selectivity [27, 28] at the seawater-catalyst interface may be the main reason for the sharp decline in the durability of InGaN/GaN NWs from 70 h in pure water to 10 h in seawater. Hence, it is important to find out a strategy for regulating the catalytic microenvironments, which can be realized by surface active-site engineering [29, 30] to tune the interaction between impurity ions and photocatalysts, thereby achieving targeted interface reactions, with operational time as long as possible. On the other hand, for the practical H_2_ production, the severe adverse reaction is unavoidable when the system operates at atmospheric pressure [8, 24]. Domen et al. deposited an amorphous SiO_2_ coating on the La_5_Ti_2_Cu_0.9_Ag_0.1_O_7_S_5_:Mg,Al/Au/BVO:Mo(h) sheet to suppress the oxygen reduction reaction [31]. The sample maintained an STH efficiency of 0.41% at near-ambient pressure and 333 K, which is about 60% of that at a low pressure of 4 kPa and 301 K. Such a situation also calls for a need to develop a new device structure that can break the high sensitivity of photocatalytic activity to background pressure and has no obvious adverse reactions.

Here, we design and construct a Ag_3_PO_4_/CdS porous microreactor chip, followed by modifications with Pt co-catalysts for improving photocatalytic performance in overall seawater splitting. It achieves an average STH efficiency of 0.81% and a maximum of 0.92% during stability testing up to 300 h, with reaction conditions at atmospheric pressure, room temperature and visible-light irradiation. For the as-prepared semiconductor chip, the type of vacancies was accurately controlled to endow the chip with reaction specificity. Specifically, the Ag vacancies on the Ag_3_PO_4_ layer repel ions in seawater to avoid detrimental competing oxidation reactions. Meanwhile, the S vacancies on the CdS layer can selectively adsorb S species and improve hydrogen evolution reactions through the tailored interaction between S species and water molecules (H_2_O). In addition to the formation of an ordered seawater-catalyst interface, a strategy for tuning the thickness of the Ag_3_PO_4_/CdS thin film within the space charge region is also adopted to ensure the efficient charge transfer from the inside of the catalyst to the interface while retaining strong redox ability. This is supported by the Kelvin probe force microscopy (KPFM) measurements, which intuitively reveal that the band bending effect in the depletion region being closely related to the heterojunction thickness. Furthermore, the multi-layer structure enables separate evolution of H_2_ and O_2_ to cleverly suppress the hydrogen and oxygen reverse recombination reaction. In a scalable hydrogen production prototype, the average H_2_ evolution rate under natural weather is 68.01 mmol h^−1^ m^−2^, even without artificial convection, temperature control and vacuum treatment. The rate can reach a maximum of 80.55 mmol h^−1^ m^−2^ at noon. Our work provides another avenue for enhancing the catalytic selectivity via engineering the catalytic microenvironment and will inspire researchers to scale up the solar H_2_ production in a different way that no longer relies solely on the spraying or bonding.

## Experimental Section

### Preparation of Ag_3_PO_4_/CdS Chips

The supporting layer (Al_2_O_3_, 99%, ϕ16 mm and 1 mm thickness, the area with and without pores each accounting for 50%), silver phosphate (Ag_3_PO_4_, 99%, granular, 8 g, Shanghai Energy Chemical) and cadmium sulfide (CdS, 99.999%, granular, 10 g, Shanghai Aladdin) were added to a coater (ZZSX-800, Beijing Instrument Factory) in turn, with the former located in coating fixtures (30 mm in width, 30 mm in length and 2 mm in height) at the top, and the film-forming materials were placed in the oxygen-free copper crucible (inner diameter: 29.5 mm) at the bottom. The pressure and temperature of the coater were set to 2.0 × 10^–3^ Pa and 200 °C, respectively. Under the above operation conditions, we turned on the ion source and then injected argon (Ar: 10 sccm) to etch the supporting layer for 6 min. After completing the premelting of Ag_3_PO_4_ using the E-Beam gun (beam current: 15 mA) for 30 s, the deposition rate was set to 0.5 nm s^−1^ until the crystal monitor controlled the completion of a 30-nm Ag_3_PO_4_ on the supporting layer. The deposition procedure of CdS on the Ag_3_PO_4_ surface is similar to that of Ag_3_PO_4_ on supporting layer while the premelting time, beam current and deposition rate were set to 10 s, 3 mA, and 2 nm s^−1^ until the crystal monitor controlled the completion of a 60-nm CdS on the Ag_3_PO_4_. During the deposition process of Ag_3_PO_4_ and CdS, oxygen (O_2_: 15 sccm) was introduced to control the vacancy concentrations. Then, the above semi-finished samples and platinum (Pt, 99.99%, 5 g, Beijing General Research Institute of Nonferrous Metals) were added to a coater (ZZS800-2/G, SDIC Nanguang Co., Ltd.) in turn, with the former located in coating fixtures (30 mm in width, 30 mm in length and 2 mm in height) at the top, and the Pt was placed in the graphite crucible (inner diameter: 29.5 mm) at the bottom. The pressure and temperature of the coater were set to 2.0 × 10^–3^ Pa and 200 °C, respectively. After completing the premelting of Pt using the E-Beam gun (beam current: 360 mA) for 10 s, the deposition rate was set to 0.1 nm s^−1^ until the crystal monitor controlled the completion of a 0.3-nm Pt on the CdS surface. After 48 h “double 85” wet-hot test (85% RH/85 °C), no detachment is observed in the scotch tape tests, indicating the reliable immobilization of the film.

### Characterization

The morphology, elemental composition and distribution of the samples were analyzed using a scanning electron microscopy (SEM, ZEISS GeminiSEM 300) operated at an accelerating voltage of 3 kV. The morphology of Pt atomic clusters was recorded using a Dimension Icon atomic force microscope (AFM). The transmission electron microscopy (TEM) was determined after focused ion beam (FIB, FEI Scios 2 HiVac). TEM, high resolution-TEM (HR-TEM), TEM-EDS and HAADF-STEM observations were carried out on a transmission electron microscopy (FEI Talos F200X G2) operated at an accelerating voltage of 200 kV. Aberration-corrected (AC)-TEM observations were carried out on a transmission electron microscopy (JEOL ARM-200F) operated at an accelerating voltage of 200 kV. Continuous-wave electron paramagnetic resonance (EPR) spectra were obtained by using an X-band (9.4 GHz) Bruker EMXplus EPR spectrometer. All measurements were carried out at 298 K. All X-Band spectra were collected over a 100 Gauss field range and 5 scans were adopted for each measurement. X-ray photoelectron spectroscopy (XPS) data were analyzed using an X-ray photoelectron spectrometer (Thermo scientific K-Alpha^+^). The X-ray diffraction (XRD) patterns were examined on a multipurpose X-ray diffraction system (SmartLab SE, Rigaku) utilizing Cu Kα radiation working at an acceleration voltage of 45 kV and a current of 200 mA. The amplitude-modulation KPFM was operated combined with a Cypher S AFM (Asylum Research, Oxford Instruments) and a HF2LI Lock-in amplifier (Zurich Instruments) in N_2_-filled glovebox. The resonance frequency ω_0_ and spring constant of AFM conducting tips are ~ 127 kHz and 5.0 N m^−1^, respectively. The absorption spectra were investigated using a UV–visible-NIR spectrophotometer (Agilent Cary 7000). The Mott-Schottky plot and electrochemical impedance spectroscopy (EIS) were recorded on the CHI760E electrochemical workstation with a standard three-electrode system. A 0.5 M Na_2_SO_4_ solution was used as the electrolyte. Fourier transform infrared spectra were studied through an infrared microscope (Thermo Scientific Nicolet iN10). Zeta potential analysis was conducted using a laser particle size analyzer (Anton Paar SurPASS 3). The salinity of natural seawater was measured by a salinometer (DLX-ART032, DELIXI). The components of natural seawater were analyzed using an inductively coupled plasma optical emission spectrometer (ICP-OES) (Agilent 5110).

### Photocatalytic Measurements (Lab)

Photocatalytic overall seawater splitting reactions were performed in a 320-mL quartz glass reactor sealed by a vacuum sealing quartz cover and a vacuum sealing rubber ring. The seawater (salinity: 33‰; Cl: 18,421.706 mg kg^−1^; Na: 9779.968 mg kg^−1^; Mg: 1208.715 mg kg^−1^; S: 811.583 mg kg^−1^; Ca: 381.820 mg kg^−1^; K: 335.505 mg kg^−1^) was collected from Changle, Fuzhou city, Fujian Province, China. In a typical time-course of seawater-splitting experiment, the reactor was purged with N_2_ flow to eliminate the air before the test, but no vacuum treatment was performed to ensure that the reaction was carried out at room pressure environment. A sample held with a fixture was put in 40 mL seawater under visible-light irradiation of a 300 W Xe lamp with a 400–780 nm cutoff filter for simultaneous evolution of H_2_ and O_2_. The temperature of the reaction solution was kept at 20 °C throughout the whole experiment. At a given interval of 1 h, 1 mL of the evolved gas was removed from the reactor and then determined using a gas chromatograph (Fuli 9790, with high-purity nitrogen as the carrier gas) equipped with a thermal conductivity detector worked at 120 °C. For OWS reactions using pure water and artificial seawater as reaction solutions, the same experimental process, reaction environment and testing method were adopted. In the long-term stability test, the photocatalytic OWS reaction was conducted using the same method every day.

Photodegradation experiments of methyl orange (MO) and rhodamine B (RhB) dyes were also performed in a 320-mL quartz glass reactor sealed by a vacuum sealing quartz cover and a vacuum sealing rubber ring. The reactor was purged with N_2_ flow to eliminate the air before the test. A sample held with a fixture was put in reaction solution (100 mL, 8 mg L^−1^ MO or 8 mg L^−1^ RhB) under visible-light irradiation of a 300 W Xe lamp with a 400–780 nm cutoff filter. The temperature of the reaction solution was kept at 20 °C throughout the whole experiment. At a given interval of 1 h, 3 mL of suspension was removed from the reactor and then filtrated through a filter membrane. The characteristic signals of MO and RhB were, respectively, determined at 464 and 553 nm by a UV–visible-NIR spectrophotometer (Agilent Cary 7000), and the concentration of the dye was obtained according to the pre-plotted calibration line. Meanwhile, 1 mL of the evolved gas was removed from the reactor and then determined using a gas chromatograph (Fuli 9790, with high-purity nitrogen as the carrier gas) equipped with a thermal conductivity detector worked at 120 °C.

### Photocatalytic Measurements (Outdoor)

Photocatalytic overall seawater splitting reactions were performed in a 2250 mL quartz glass reactor sealed by a vacuum sealing quartz cover and a vacuum sealing rubber ring. The reactor was purged with N_2_ flow to eliminate the air before the test, but no vacuum treatment is performed to ensure that the reaction was carried out at room pressure environment. 25 samples held with fixtures were put in 500 mL seawater under natural sunlight for simultaneous evolution of H_2_ and O_2_. There is no external support such as artificial convection and temperature control. At a given interval of 1 h, 1 mL of the evolved gas was removed from the reactor and then determined using a gas chromatograph (Fuli 9790, with high-purity nitrogen as the carrier gas) equipped with a thermal conductivity detector worked at 120 °C.

## Results and Discussion

### Catalyst Preparation and Characterizations

**Fig. 1:**
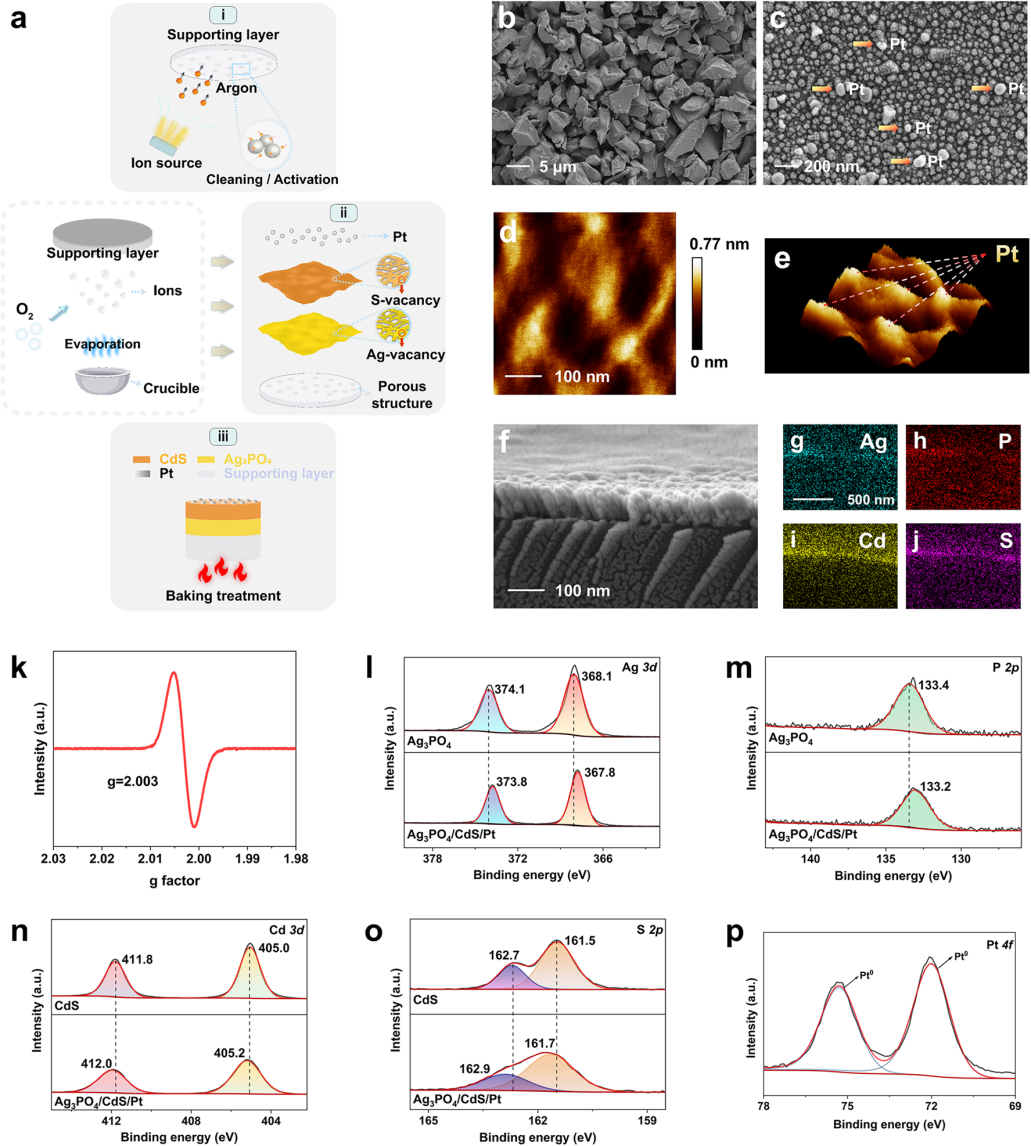
Catalyst synthesis and characterization. **a** Schematic of the preparation procedure of Ag_3_PO_4_/CdS porous microreactor chip. **b** Low and **c** high magnification SEM images of Ag_3_PO_4_/CdS porous microreactor chip. **d** Two-dimensional and **e** three-dimensional AFM images of Pt atomic clusters on the CdS. **f** Cross-sectional SEM image of Ag_3_PO_4_/CdS porous microreactor chip. **g–j** EDS cross-sectional mapping images of Ag_3_PO_4_/CdS porous microreactor chip. **k** EPR pattern of Ag_3_PO_4_/CdS porous microreactor chip. Compared XPS spectra of **l** Ag *3d* and **m** P *2p* orbits of Ag_3_PO_4_ film and Ag_3_PO_4_/CdS porous microreactor chip. Compared XPS spectra of **n** Cd *3d* and **o** S *2p* orbits of CdS film and Ag_3_PO_4_/CdS porous microreactor chip. **p** High-resolution XPS spectra of Pt *4f* orbit of Ag_3_PO_4_/CdS porous microreactor chip

Ag_3_PO_4_/CdS porous microreactor chip photocatalysts were prepared by electron beam evaporation technology. The photocatalyst consists of CdS as H_2_-evolving component and Ag_3_PO_4_ as O_2_-evolving component grown layer by layer on a supporting layer, with Pt loaded onto the CdS surface acted as the water-reduction co-catalyst. Figure 1a describes the preparation process of the sample. We turned on the ion source and then injected argon to etch the supporting layer, achieving the effect of cleaning and activation. The film-forming materials (Ag_3_PO_4_, CdS and Pt) placed in the crucible were sequentially heated by the electron beam, and then the condensible vapor was transformed from a gas or plasma phase into a solid phase on the supporting layer. During the process, O_2_ was introduced through the gas inlet to control the vacancy concentration. After three stages of nucleation, growth and film formation, the preparation was completed under the help of baking treatment (see Fig. S1 for photographs of samples).

The thickness of Ag_3_PO_4_ and CdS layers was examined by a three-dimensional optical profiler (Zygo Zegage) to be about 30 and 60 nm, respectively (Fig. S2). As seen in the SEM images (Fig. 1b and S3), irregular pores with size ranged from 1 to 10 μm were scattered around the catalytic reaction unit. Not only is it conducive to the adsorption of H_2_O, but the discontinuity renders the catalyst with reactive surface. The enlarged SEM image in Fig. 1c showed the distribution of Ag_3_PO_4_/CdS thin film on the supporting layer, with Pt atomic clusters of different sizes dispersed on the top of the film. AFM images can confirm the successful deposition of Pt with an average thickness of about 0.3 nm (Fig. 1d, e). The cross-sectional SEM image (Fig. 1f) presented that Ag_3_PO_4_ and CdS layers, with a clear boundary, were orderly deposited on the supporting layer. Note that a good interface and continuity existed between Ag_3_PO_4_ and CdS layers would be an essential prerequisite for building energetic built-in electric field. Energy-dispersive X-ray spectroscopy (EDS) elemental mapping demonstrated the existence of Ag, P, Cd, S, and Al elements (Figs. 1g−j and S4). In the phase transition process, the film-forming materials entered the supporting layer with unique porous structure. Hence, the first four elements were also present on the supporting layer portion. The TEM-EDS elemental mapping of Ag, P, Cd, and S elements is also provided (Fig. S5). Owing to the introduction of S and Ag vacancies, the molar ratio of Cd–S and Ag–P–O are, respectively,

1:0.91 and 2.7:1:4.4 (Fig. S6). A distinct EPR signal at g = 2.003 is evidence of the existence of S vacancies (Fig. 1k and S7) [32]. There is further evidence for defective structures in HR-TEM and AC-TEM images (Fig. S8). Meanwhile, the chemical compositions and elemental chemical states of Ag_3_PO_4_ film, CdS film and Pt-loaded Ag_3_PO_4_/CdS (denoted as Ag_3_PO_4_/CdS/Pt) composite were confirmed by the XPS (Figs. S9 and S10). For Ag_3_PO_4_ film, the peaks located at 368.1 and 374.0 eV can be assigned to Ag *3d*_*5/2*_ and Ag *3d*_*3/2*_ of Ag^+^ (Fig. 1l), respectively, and the binding energy peak centered at 133.4 eV corresponds to P *2p* coming from PO_4_^3−^ (Fig. 1m). As for CdS film, two peaks with the binding energies of 405.0 and 411.8 eV can be attributed to Cd *3d*_*5/2*_ and Cd *3d*_*3/2*_ of Cd^2+^ (Fig. 1n), respectively, while the peaks of S *2p* at 161.5 and 162.7 eV showed that S^2−^ existed in CdS (Fig. 1o). After coupling CdS and Pt with Ag_3_PO_4_, Cd *3d* and S *2p* shift to the high binding energy direction by 0.2 eV, and Ag *3d* and P *2p* shift to the low binding energy direction by 0.2–0.3 eV, indicating that the electrons are transferred from CdS to Ag_3_PO_4_. From the high-resolution XPS spectra of Pt *4f* (Fig. 1p), it can be inferred that Pt is in a metallic state [33]. Besides, the body-centered cubic structure Ag_3_PO_4_ (JCPDS NO. 06-0505) and the hexagonal phase of CdS (JCPDS NO. 41–1049) were observed in Ag_3_PO_4_/CdS thin film by grazing incidence X-ray diffraction (GIXRD) analysis (Fig. S11).

### Visualization of Energy Band Alignment in Space Charge Region

**Fig. 2:**
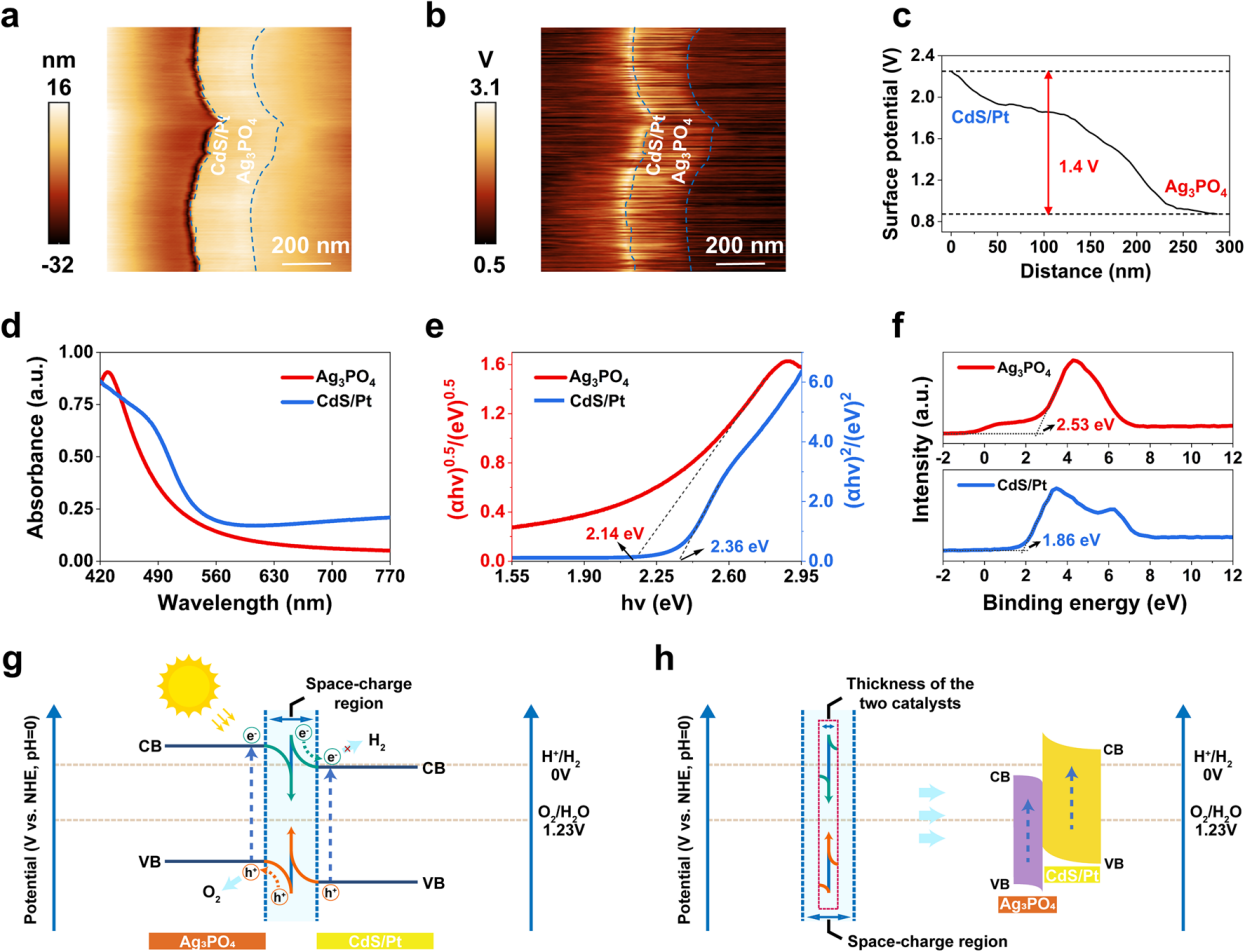
Visualization of energy‐band bending. **a** AFM topography and **b** surface potential image at the interface of Ag_3_PO_4_/CdS/Pt heterojunction measured by KPFM. **c** The surface potential profile at the interface of CdS/Pt and Ag_3_PO_4_ layers. **d** UV–visible diffuse reflectance spectra of Ag_3_PO_4_ and CdS/Pt thin films. **e** (*αhν*)^1/2^ versus (*hν*) for Ag_3_PO_4_ and (*αhν*)^2^ versus (*hν*) for CdS/Pt. **f** XPS VB spectra of Ag_3_PO_4_ and CdS/Pt thin films. **g** Reduction in redox ability caused by band bending to make the sample without the capability of overall water splitting. **h** Schematic diagram of the mechanism of overall water splitting for Ag_3_PO_4_/CdS/Pt heterojunction with thickness smaller than the space charge region

It is necessary to further prove that a heterojunction is formed between the Ag_3_PO_4_ and CdS. KPFM visualized the energy-band alignment near the interface of Ag_3_PO_4_/CdS/Pt nanocomposites, with a thickness not exceeding the space charge region width (approximately 354 nm, see discussions in Note S1). According to the analysis of the topography and surface potential (Fig. 2a–c), the contact potential difference (CPD) of CdS/Pt is 1.4 V lower than that of Ag_3_PO_4_. It leads to the electron transfer from CdS to Ag_3_PO_4_ and induces an upward band bending in CdS and a downward band bending in Ag_3_PO_4_. More importantly, there is a corresponding band bend for each thickness-related position, but for the first time no flat band is observed, which is quite different from the typical heterojunction. Thinner Ag_3_PO_4_/CdS/Pt nanocomposite was prepared to further confirm this phenomenon. The contrast of CPD is reduced to 0.20 V and further to 0.12 V under light excitation (Fig. S12), indicating the photogenerated charge transferring to the surface. The analysis above confirmed the successful preparation of heterojunction with thickness smaller than the space charge region. And the controllable regulation of band bending can be achieved by tuning the thickness of heterojunction [34], thereby endowing the sample with different photocatalytic driving forces. Guided by this mechanism, the optimal thickness of Ag_3_PO_4_/CdS/Pt heterojunction has been determined (30 nm Ag_3_PO_4_ and 60 nm CdS). As shown in Fig. 2d, Ag_3_PO_4_ film exhibits efficient optical absorption in the visible-light region with an absorption edge around 570 nm, while that of CdS/Pt film is near 550 nm, ensuring the production of sufficient excitons. According to the transmittance spectrum of CdS/Pt thin film, the CdS/Pt and Ag_3_PO_4_ layers occupy, respectively, 65% and 35% of the useful visible light (Fig. S13). As determined from the Tauc equation [35], the bandgaps of the Ag_3_PO_4_ and CdS/Pt were calculated to be 2.14 and 2.36 eV, respectively (Fig. 2e, see discussions in Table S1 and Note S2). As revealed by the XPS valence band (VB) spectra and Mott-Schottky plots, the VB and conduction band (CB) potentials of Ag_3_PO_4_ film were calculated as 2.53 and 0.39 eV, with a bandgap of 2.14 eV, and that of CdS/Pt film were 1.86 and -0.51 eV with a bandgap of 2.37 eV (Fig. 2f and S14). The bandgaps obtained by these two methods are consistent.

On basis of above analyses, we proposed the schematic of reaction mechanism of Ag_3_PO_4_/CdS/Pt heterojunction for photocatalytic OWS. The generation and transfer process of photogenerated charges can be described as follows [36]: Ag_3_PO_4_/CdS/Pt heterojunction produced nonequilibrium electrons and holes under light irradiation. The synergistic effect of Coulomb attraction and built-in electric field facilitates the electron transfer from CB of Ag_3_PO_4_ to VB of CdS/Pt, resulting in the annihilation of weak carriers at the CdS/Ag₃PO₄ interface. High-energy electrons and holes, meanwhile, are retained in CB of CdS/Pt and VB of Ag_3_PO_4_. The efficient charge transfer in Ag_3_PO_4_/CdS/Pt heterojunction was also confirmed by EIS (Fig. S15). In the case of large thickness, however, the band bending caused by the difference in Fermi level results in CdS/Pt not being able to reduce H_2_O to H_2_, because of its CB being more positive than the reduction potential of H_2_O/H_2_ (Fig. 2g). In light of this phenomenon, tuning the thickness of Ag_3_PO_4_ and CdS layers to 30 and 60 nm within the space charge region effectively avoids the band bending (Fig. 2h). As revealed by the XPS VB analyses of Ag_3_PO_4_/CdS/Pt heterojunctions (Fig. S16), the VB potential of Ag_3_PO_4_ is slightly negative shifted to 2.39 eV after heterojunction formation, and that of CdS/Pt is slightly positive shifted to 2.09 eV. Thus, the oxidation ability of Ag_3_PO_4_ and the reduction ability of CdS/Pt are retained to initiate the O_2_ and H_2_ evolution reactions, respectively. Besides, the band structure diagram showing the variations of the Fermi level in different states was also analyzed to help understand the reaction mechanism of Ag_3_PO_4_/CdS chip (Fig. S17).

### Photocatalytic Overall Seawater Splitting

**Fig. 3:**
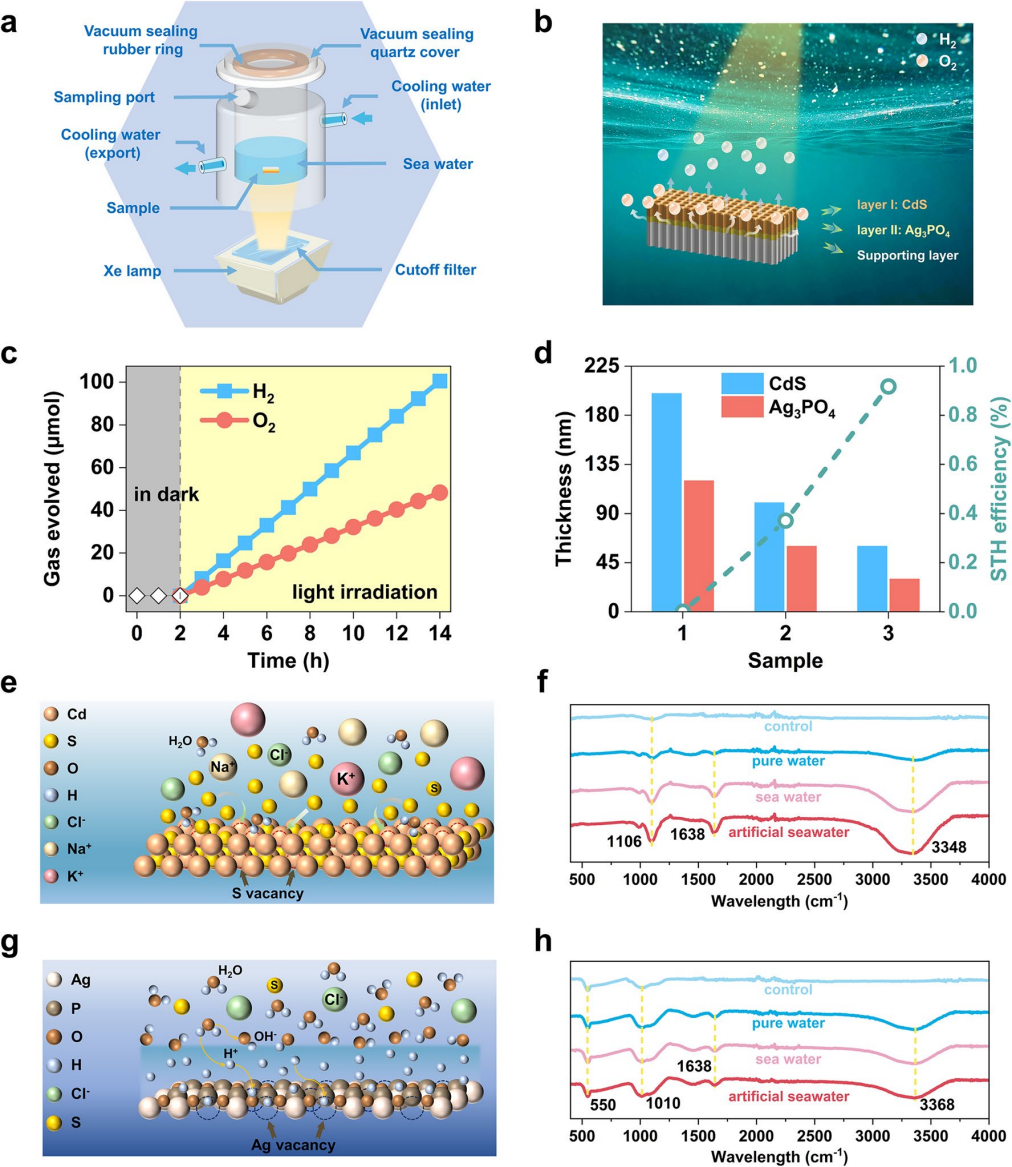
Porous microreactor chip for seawater splitting and ordered seawater-catalyst interface. **a** Schematic illustration of the reaction system. **b** Facile separation of H_2_ and O_2_. **c** Typical time course of H_2_ and O_2_ production. **d** The overall seawater-splitting performance of Ag_3_PO_4_/CdS porous microreactor chips with different thickness of photocatalytic layers. Schematic showing the selective adsorption of water and ions on the **e** CdS and **g** Ag_3_PO_4_. FTIR spectra of **f** CdS and **h** Ag_3_PO_4_ thin films

Here, a harsh reaction environment at atmospheric pressure and room temperature was adopted for performing OWS in seawater (Fig. 3a). Under the built-in electric field, electrons migrate to the surface of CdS to reduce H_2_O, while holes migrate to the surface of Ag_3_PO_4_ to oxidize H_2_O. Therefore, H_2_ come out of the CdS, while O_2_ come out of the Ag_3_PO_4_ (Fig. 3b). The separation of H_2_ and O_2_ in space avoids their direct contact and recombination. As a result, simultaneous evolution of H_2_ and O_2_ with a stoichiometric ratio close to 2:1 was observed (Fig. 3c). Small amount of produced O_2_ will dissolve in seawater. The production rates of H_2_ and O_2_, in a typical time-course experiment under the visible-light irradiation (40 mW cm^−2^), reached 8.37 and 4.02 μmol h^−1^, respectively, and the measured STH conversion efficiency was 0.92% with an apparent quantum yield (AQY) of 12.26% at 420 nm (Fig. 3c, see discussions in Notes S3 and S4). Comparison of our AQY value with those of other Ag_3_PO_4_-based and CdS-based photocatalysts is provided (Table S2). Under the simulated sunlight irradiation (AM 1.5, 100 mW cm^−2^), the STH efficiency was 0.90%. In the reaction stage, dense bubbles appeared on the sample but no gas was detected in the dark, indicating that the reaction is indeed a photo-induced process (Fig. S18). To further verify the origin of oxygen in the test, 3.5 wt% NaCl solution made of H_2_^18^O (pH = 7.1) was used for isotope labeling experiments. The molar content of the evolved ^18^O_2_ is 94%, proving that evolved O_2_ is generated by overall seawater splitting reaction (Fig. S19). Further, it is necessary to determine the thickness effect of Ag_3_PO_4_/CdS thin films on the photocatalytic activity. As depicted in Fig. 3d, the sample with Ag_3_PO_4_/CdS at 120/200 nm cannot split seawater into H_2_ and O_2_, while the H_2_ production rate reaches the maximum when the thickness of Ag_3_PO_4_ and CdS is, respectively, 30 and 60 nm. The results confirm that exploring the thickness-dependent photocatalytic performance is valuable and critical for efficient overall seawater splitting.

### Ordered Water/Catalyst Interface

Considering the performance differences of photocatalysts operating in different environments, the Fourier transform infrared (FTIR) spectra analysis was employed to investigate the dynamic process of water molecules at the water/catalyst interface. The samples adopted four treatments, including (1) no treatments (control), (2) immersed in pure water, (3) immersed in seawater (pH = 8.0) and (4) immersed in artificial seawater (pH = 12.0), i.e., natural seawater with added 0.01 mol L^−1^ Na_2_S. In the FTIR spectra of CdS film (Fig. 3f), the peak at 1106 cm^−1^ can be assigned to the vibration of Cd − S bond [37], and the peaks at 1638 and 3348 cm^−1^ can be attributed to the O − H bending and stretching vibrations, respectively. Comparing the FTIR spectra of CdS film immersed in pure water and seawater, it is noticeable that the intensity of Cd − S and O − H bonds are noticeably higher on the pink curve (seawater) than on the dark blue curve (pure water). Abundant S vacancies with uniform electronic state and coordination structure formed a stable combination with the S species that have matching chemical valence owing to the electron orbital interaction [38], along with a significant increase in the concentration of H_2_O at the seawater-catalyst interface relying on bonding interaction between sulfides and H_2_O (Fig. 3e). Meanwhile, the formation of mass-transfer bridge from CdS to sulfide then to H_2_O enables the efficient transfer of photogenerated carriers from the inside out. To verify the role of S species in establishing ordered interfacial water behavior, the FTIR spectrum of CdS film immersed in artificial seawater was measured. The intensity of Cd − S and O − H bonds further increases (red curve), implying that more sulfides and water molecules are adsorbed on the CdS. In the FTIR spectra of Ag_3_PO_4_ film (Fig. 3h), the similar characteristic peaks at 550, 1010, 1638, and 3368 cm^−1^ were observed in four curves. The former two can be assigned to the P − O stretching vibrations of PO_4_^3−^ [39], and the latter two can be attributed to the O − H bending and stretching vibrations, respectively. All characteristic peaks remain almost unchanged after soaking in pure water, seawater or artificial seawater, confirming that the Ag_3_PO_4_ film possesses excellent catalytic selectivity for all impurity ions in seawater. The Ag vacancies hinder various ions but adsorb H_2_O, inducing a negative zeta potential of − 211 mV on the slipping plane of Ag_3_PO_4_. Thanks to the electrostatic interaction, Ag_3_PO_4_ further repels the ions outside the stern layer, eliminating the occurrence of harmful competing oxidation reactions (Fig. 3g). Photocatalytic OWS in pure water, seawater and artificial seawater were carried out and the STH efficiency achieved in 12 h tests were, respectively, 0.71%, 0.92% and 1.72% (Figs. 3c and S20, Table S3). These differences clearly showed the feasibility of a vacancy-control strategy for improving the photocatalytic activity of Ag_3_PO_4_/CdS porous microreactor chips operating in high salinity environments. To further demonstrate that the selectivity of chips for various impurities stems from vacancy regulation rather than thermodynamic requirement, the photodegradation experiment for the 8 mg L^−1^ MO and RhB solutions were carried out. Although the photogenerated holes in the VB of Ag_3_PO_4_ with strong oxidation ability are far enough to oxidize these organic pollutants, both MO and RhB dyes did not degrade in the 8 h tests. The corresponding STH efficiency were, respectively, 0.66% and 0.63%, indicating that the sample also possesses catalytic selectivity for common dyes (Fig. S21). Therefore, the proposed vacancy-control strategy is expected to expand the application range of light-driven water splitting to a range of open-water sources.

### Performance Stability and Scalability

**Fig. 4:**
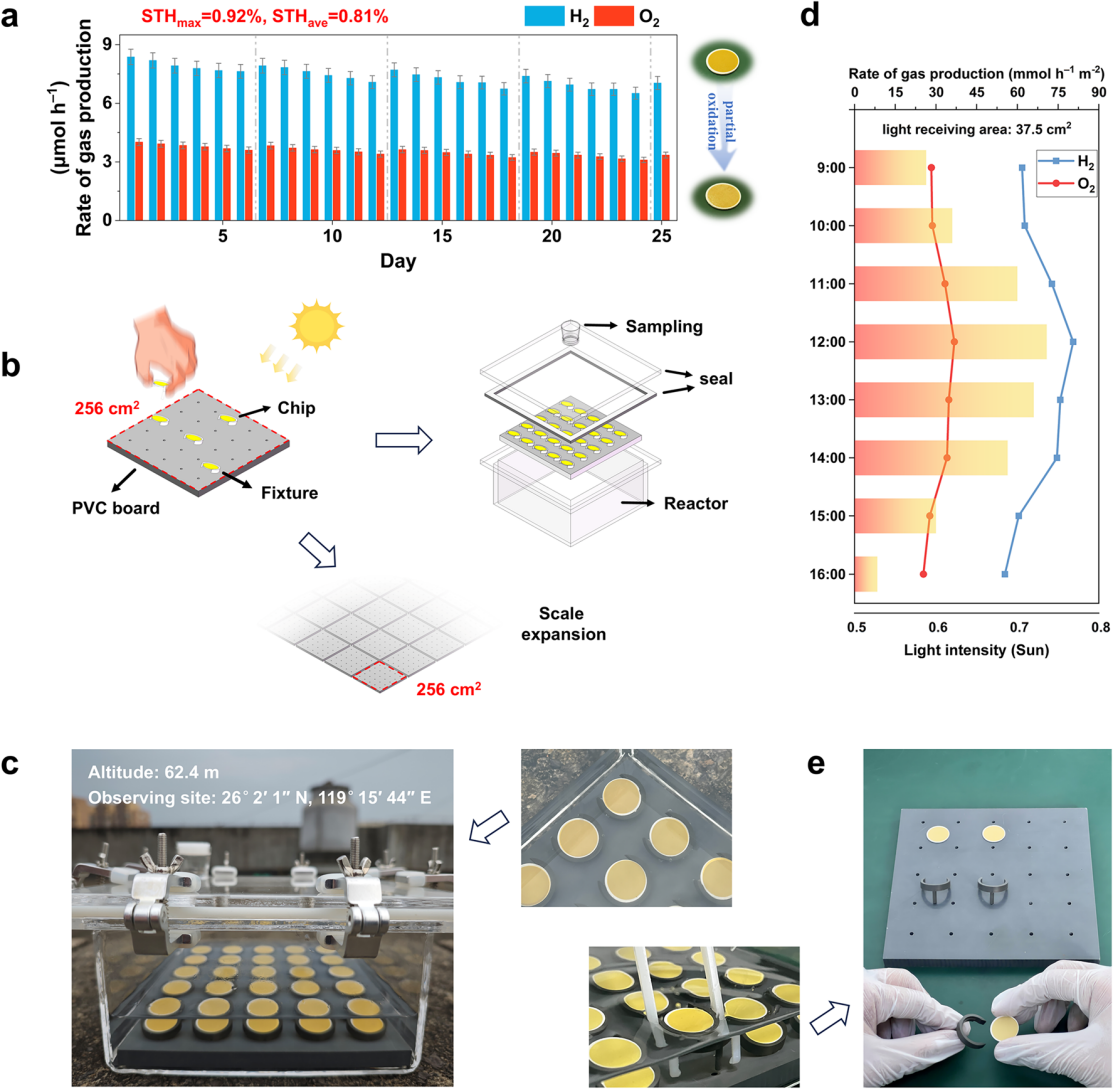
Recycle H_2_/O_2_ generation property and large-scale hydrogen production prototype. **a** Stability test of as-prepared Ag_3_PO_4_/CdS porous microreactor chip photocatalysts. Each cycle is 12 h. **b** The design and **c** optical image of an outdoor set-up. **d** Outdoor set-up for H_2_ and O_2_ evolution via the seawater splitting reaction. **e** Facile disassembly and recycling

Based on the analysis above, a rapid carrier transport from the inside of the catalyst to the water molecules is formed by tuning heterojunction thickness and targeted vacancy regulation. It contributed to efficient photocatalytic overall seawater splitting within a single cycle. However, considering the difficulty but importance of long-term operation of photocatalysts in seawater environments, the photocatalytic stability of the chip was investigated (Fig. 4a and Table S4). During the 25-day cycles, the photoactivity remained relatively constant, which means that the Ag_3_PO_4_/CdS porous microreactor chip can stably achieve OWS reaction in seawater. The samples were washed with pure water after every six cycles. The good stability of the chip within 300 operating hours is far more durable than the previously reported overall seawater-splitting system with the durability of 12 h at most (Table S5). The structure of thin film deposited on the reaction unit with vacancies regulation effectively avoids the flocculation effect and detrimental competing oxidation reaction. Continuous gas evolution can prevent scaling issues. All these characteristics greatly extend the durability of the catalyst. The almost unchanged morphology, EDS, XPS and XRD spectra after the long-term tests further proved that the sample are highly stable in seawater (Fig. S22). A 16 percent decline in activity from the first test to the twenty-fifth test probably results from decrease in S vacancies caused by oxidation, and its signal intensity decreases by about 14% (Fig. S23). The correlation between activity and vacancy concentration was further determined by tracking changes in activity of freshly prepared samples stored under different conditions (Fig. S24). Once a way is found to enhance the stability of vacancies on the film surface or restore vacancies, the durability of Ag_3_PO_4_/CdS porous microreactor chips will be further improved.

The scaleup of the photocatalytic OWS system is a prerequisite for practical applications. We fixed the chips with a custom-designed fixture and arrayed them on a polyvinyl chloride (PVC) board to assemble a large-scale hydrogen production prototype with an area of 256 cm^2^ (Fig. 4b). There is an inclination angle of 20 degrees between the chip and the plane of PVC board, which not only compensates for the light loss caused by unnormal incidence of sunlight, but also ensures that the resulting gas escapes quickly from the surface of the catalyst. Under natural sunlight, this prototype showed a highest hydrogen evolution rate of 80.55 mmol h^−1^ m^−2^ at 12:00 noon (~ 0.72 sun) with an average hydrogen evolution rate of 68.01 mmol h^−1^ m^−2^ throughout the test (Figs. 4c, d and S25), without any external support such as artificial convection, temperature control and vacuum treatment. After the experiment, the assembled system can be easily disassembled to recycle chips (Fig. 4e). In principle, expanding the Ag_3_PO_4_/CdS porous microreactor chip to 1 m^2^ is expected to produce over 0.54 mol of H_2_ per day, and the scale can be further expanded because it is easy to assemble (Fig. 4b). Meanwhile, a circular glass reactor was designed to meet different terrain requirements (Fig. S26).

## Conclusions

We have designed a multilayer porous semiconductor chip and systematically demonstrated its superiority over conventional photocatalysts in overall seawater splitting. Vacancy control endowed the interface reaction with specificity; heterojunction design enabled efficient carrier transport, and multi-layer structure ensured convenient gas separation. Therefore, even in harsh environment of atmospheric pressure, room temperature and high salinity, the Ag_3_PO_4_/CdS porous microreactor chip exhibited a high STH efficiency of 0.81% in a 300-h test. The chip's performance stability is at least one order of magnitude larger than that of most of recently reported photocatalysts for overall seawater splitting. As an effort to scaleup, we constructed a 256 cm^2^ hydrogen production prototype, which showed a H_2_ production rate of 68.01 mmol h^−1^ m^−2^, with potentials for further expansion. The greatly enhanced stability and scalability for the chips demonstrated in this work offer a promising route to promote the practicality of technologies in light-driven seawater splitting.

## Supplementary Information

Below is the link to the electronic supplementary material.

Supplementary file1 (DOCX 30846 kb)
