# Supplementary material for: Porous Microreactor Chip for Photocatalytic Seawater Splitting over 300 Hours at Atmospheric Pressure
Source: Nanomicro Lett. 2025 Mar 17;17:188. doi: 10.1007/s40820-025-01703-6 (PMC11914653; doi:10.1007/s40820-025-01703-6)
Supplement: Supplementary file 1 — Supplementary file1 (DOCX 30846 kb) [file 40820_2025_1703_MOESM1_ESM.docx]

Supporting Information for

**Porous Microreactor Chip for Photocatalytic Seawater Splitting over 300 Hours at Atmospheric Pressure**

Desheng Zhu^1, #^, Zhipeng Dong^1, #^, Chengmei Zhong^2^, Junhong Zhang^2^, Qi Chen^3^, Ni Yin^3^, Wencheng Jia^1^, Xiong Zheng^1^, Fengzai Lv^2, *^, Zhong Chen^1, 5^, Zhenchao Dong^4, *^, Wencai Huang^1, *^

^1^ Department of Electronic Engineering, School of Electronic Science and Technology, Xiamen University, Xiamen 361005, P. R. China

^2^ Fuzhou Fuzhi Photocatalysis Research Center, Fuzhou 350007, P. R. China

^3^ i-Lab, CAS Key Laboratory of Nanophotonic Materials and Devices, Suzhou Institute of Nano-Tech and Nano-Bionics, Chinese Academy of Sciences, Suzhou 215123, PR China

^4^ Hefei National Research Center for Physical Sciences at the Microscale and CAS Center for Excellence in Quantum Information and Quantum Physics, University of Science and Technology of China, Hefei 230026, P. R. China

^5^ State Key Laboratory of Physical Chemistry of Solid Surfaces, Xiamen University, Xiamen 361005, P. R. China

*^#^* Desheng Zhu and Zhipeng Dong contributed equally to this work

* Corresponding authors. E-mail: [fengzailv@163.com](mailto:fengzailv@163.com) (Fengzai Lv); [zcdong@ustc.edu.cn](mailto:zcdong@ustc.edu.cn) (Zhenchao Dong); [huangwc@xmu.edu.cn](mailto:huangwc@xmu.edu.cn) (Wencai Huang)

**Note S1 Calculation method and results of space charge region**

The space charge region width d can be calculated according to the following equations:

|  | $d_{1}=\sqrt{\frac{2\varepsilon_{1}\varepsilon_{2}N_{2}V_{D}}{qN_{1}(\varepsilon_{1}N_{1}+\varepsilon_{2}N_{2})}}$ | (S1) |
| --- | --- | --- |
|  | $d_{2}=\sqrt{\frac{2\varepsilon_{1}\varepsilon_{2}N_{1}V_{D}}{qN_{2}(\varepsilon_{1}N_{1}+\varepsilon_{2}N_{2})}}$ | (S2) |

Here, d_1_ and d_2_ are the depletion region widths of different semiconductors constructing the heterojunction, ε_1_ and ε_2_ are the dielectric constants of different semiconductors, N_1_ and N_2_ are the impurity concentrations of different semiconductors and V_D_ is the built-in potential difference. Bring in the relevant parameters of Ag_3_PO_4_/CdS/Pt heterojunction into the above equations, the calculation results are as follows:

|  | $\begin{matrix} d_{1}=\sqrt{\frac{2\varepsilon_{1}\varepsilon_{2}N_{2}V_{D}}{qN_{1}(\varepsilon_{1}N_{1}+\varepsilon_{2}N_{2})}} \\ =\sqrt{\frac{2\times5.520\times8.85\times10^{-14}\times3.197\times8.85\times10^{-14}\times1\times10^{16}\times1.4}{1.602\times10^{-19}\times1\times10^{16}\times\left(5.520\times8.85\times10^{-14}\times1\times10^{16}+3.197\times8.85\times10^{-14}\times1\times10^{16}\right)}} \\ \approx177\mathrm{nm} \end{matrix}$ | (S3) |
| --- | --- | --- |
|  | $d_{1}=\text{d}_{2},d=d_{1}+\text{d}_{2}=354\mathrm{nm}$ | (S4) |

**Note S2 E_g_, E_VB_ and E_CB_ determination**

The band gap could be calculated according to the Tauc equation [S1, S2]:

|  | $\left(\alpha h\nu\right)^{1/n}=\beta(h\nu-E_{g})$ | (S5) |
| --- | --- | --- |

where *α*, *h*, *ν*, *β* and *Eg* are, respectively, absorbance coefficient, Planck constant, light frequency, proportionality constant and band gap, while term n depends on the nature of the transition in a semiconductor. For the CdS as a typical direct transition semiconductor, the value of n is 1/2, the *E_g_* of the CdS can be obtained by plotting (*hν* and (*αhν*)^2^). As an indirect transition semiconductor, the value of n for the Ag_3_PO_4_ is 2, thus the *E_g_* of the Ag_3_PO_4_ can be obtained by plotting (*hν* and (*αhν*)^0.5^). As shown in Fig. 2e, the *E_g_* of the as-prepared CdS thin film is 2.36 eV and that of the as-prepared Ag_3_PO_4_ thin film is 2.14 eV.

The VB and CB level (*E_VB_* and *E_CB_*) could be calculated according to the following equations:

|  | $E_{CB}=\chi-E_{e}-0.5E_{g}$ | (S6) |
| --- | --- | --- |
|  | $E_{VB}=E_{CB}+E_{g}$ | (S7) |

in which, *χ* represents the absolute electronegativity of the semiconductor (Table S1) and *E_e_* is a constant (4.5 eV) representing the Fermi level of a normal hydrogen electrode relative to the vacuum level at 25 °C. Therefore, the *E_CB_* and *E_VB_* of as-prepared CdS thin film are calculated to be -0.50 eV and 1.86 eV, whereas that of the as-prepared Ag_3_PO_4_ thin film are 0.39 eV and 2.53 eV.

**Note S3 Calculation method and results of STH efficiency**

For a typical time-course of visible-light-driven photocatalytic seawater-splitting experiment, the STH efficiency could be calculated from the following equation [S3, S4]:

|  | $\mathrm{STH}=R_{H_{2}}\times\frac{\Delta G}{P\times S}= \frac{8.37\times10^{-6}\times237\times10^{3}}{3600\times400\times1.5\times10^{-4}}\times100\%=0.92\%$ | (S8) |
| --- | --- | --- |

in which, *R_H2_* and *∆G* are the rate of H_2_ production and the Gibbs free energy of overall water splitting reaction. The effective irradiation region (S) of the Ag_3_PO_4_/CdS porous microreactor chip is about 1.5 cm^2^. *P* is the average light intensity of irradiation incident on the sample determined by a solar energy meter, which is controlled at 40 mW cm^-2^. In the 25-day cycle test, the STH efficiency of Ag_3_PO_4_/CdS porous microreactor chip photocatalyst is calculated using the same method, and the results are shown in Fig. 4a and Table S4. For the OWS reaction using pure water and artificial seawater as reaction solution, the calculation method of STH efficiency is the same, and the results are shown in Fig. S20 and Table S3.

Subsequently, the chip is illuminated by a solar light simulator (AM 1.5, 100 mW cm ^-2^). The sample is tested for 12 hours. The H_2_ production rate is 20.52 μmol h^-1^, and the STH conversion efficiency is calculated to be 0.90%.

**Note S4 Calculation method and results of AQY value**

Here, the photocatalytic seawater-splitting experiment is carried out using the same experimental set-up described in the main text except for the use of a 420 ± 20 nm bandpass filter to provide the monochromatic light. The incident energy flux to the sample is controlled at 24.7 W m^-2^ and the produced H_2_ in 12 hours is 17.22 μmol. The AQY value at 420 nm for H_2_ evolution could be calculated according to the following equations [S3, S5, S6]:

|  | $N=\frac{E\times\lambda}{h\times c}=\frac{24.7\times1.5\times10^{-4}\times12\times3600\times420\times10^{-9}}{6.626\times10^{-34}\times3\times10^{8}}=3.382\times10^{20}$ | (S9) |
| --- | --- | --- |
|  | $\begin{matrix} \mathrm{AQY}(\%)=\frac{\mathrm{number} \mathrm{of} \mathrm{produced} \mathrm{H}_{2} \mathrm{molecules}\times4}{\mathrm{number} \mathrm{of} \mathrm{incident} \mathrm{photons}}\times100\% \\ =\frac{\text{n}\times N_{A}\times4}{N}\times100\% \\ =\frac{17.22\times10^{-6}\times6.02\times10^{23}\times4}{3.382\times10^{20}}\times100\% \\ =12.26\% \end{matrix}$ | (S10) |

**Supplementary Figures and Tables**


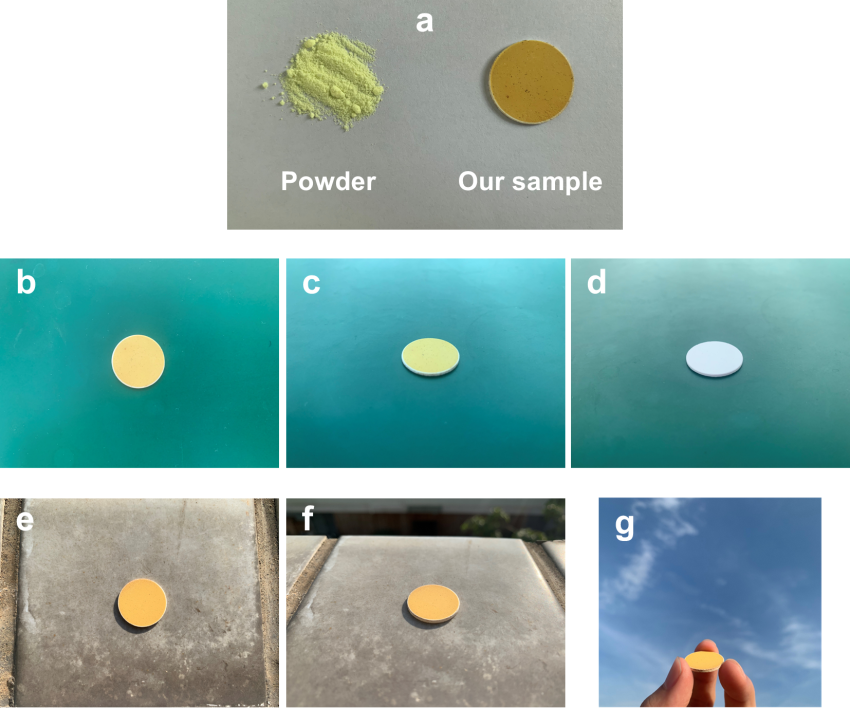


**Fig. S1** Optical images of the Ag_3_PO_4_/CdS porous microreactor chip photocatalysts. The sample has a bilayer structure, with the upper layer being a photocatalytic layer composed of Ag_3_PO_4_ and CdS thin films, and the lower layer being a supporting layer composed of alumina. (**a**) Structure comparison between photocatalyst powder and Ag_3_PO_4_/CdS porous microreactor chip photocatalyst. An advantage of our sample over traditional photocatalyst powder is that there is no flocculation effect when working in seawater, making it easy to recover. On the other hand, the sample could be stored for a long time under ambient conditions without observable changes. (**b, c**) sample photos taken indoors from different angles. (**d**) photo of supporting layer. (**e**–**g**) sample photos taken outdoors from different angles


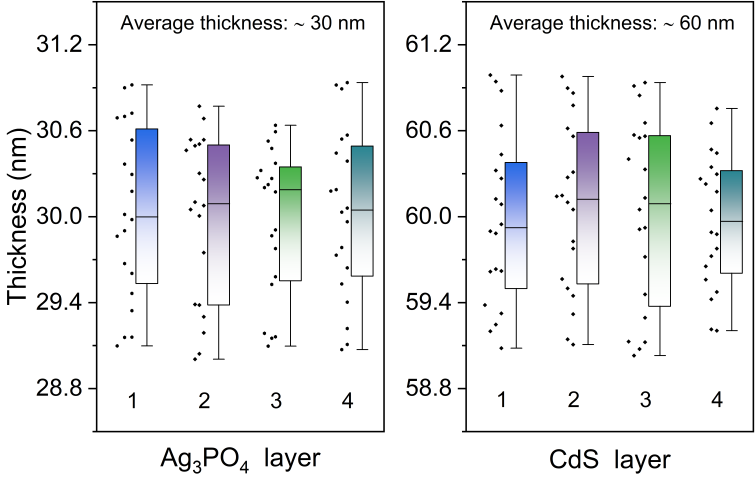


**Fig. S2** Thickness statistics for Ag_3_PO_4_ and CdS layers of Ag_3_PO_4_/CdS porous microreactor chip photocatalysts in four batches. There are 20 chips for each batch, with the line around the center representing the mean value


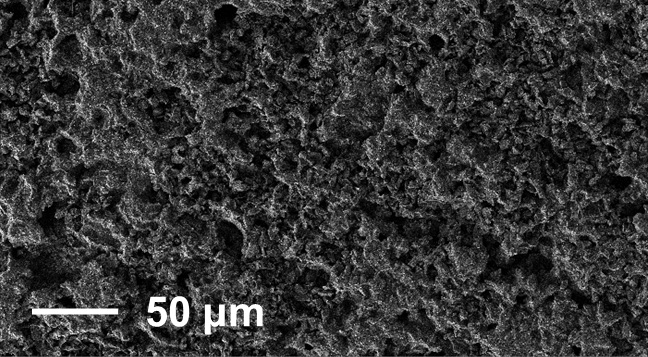


**Fig. S3** SEM image of a supporting layer with a porous structure


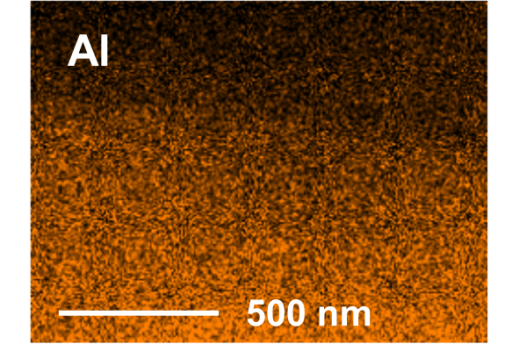


**Fig. S4** EDS elemental mapping of Ag_3_PO_4_/CdS porous microreactor chip photocatalyst (aluminum element)


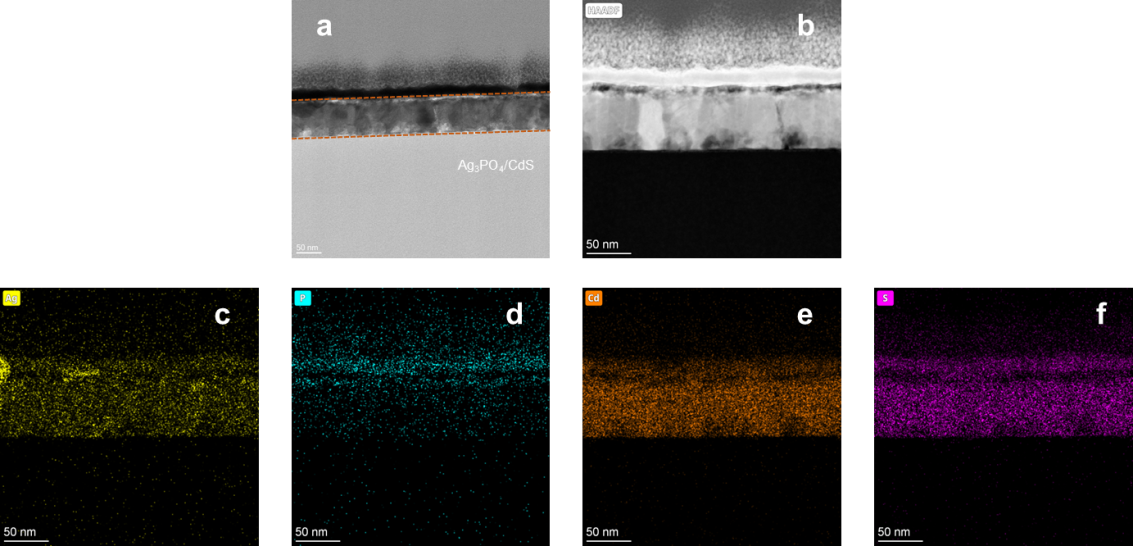


**Fig. S5** (**a**) TEM and (**b**) HAADF-STEM images of Ag_3_PO_4_/CdS chip and corresponding elemental mapping of Ag (**c**), P (**d**), Cd (**e**) and S (**f**) elements


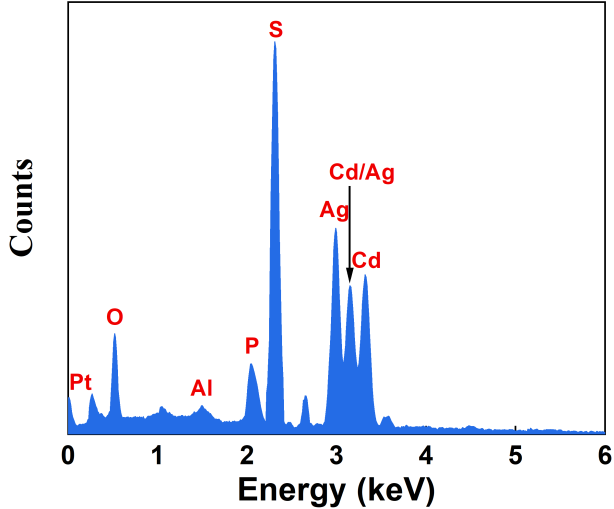


**Fig. S6** EDS spectrum of Ag_3_PO_4_/CdS porous microreactor chip photocatalyst. All elements of Ag, P, O, Cd, S, Pt and Al can be found


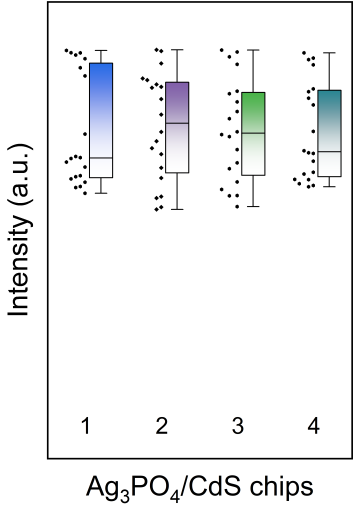


**Fig. S7** Statistical analysis of EPR signals for Ag_3_PO_4_/CdS chips in four batches. There are 20 chips for each batch, with the line around the center representing the mean value. The sulfur vacancy concentration of all chips exhibit good uniformity


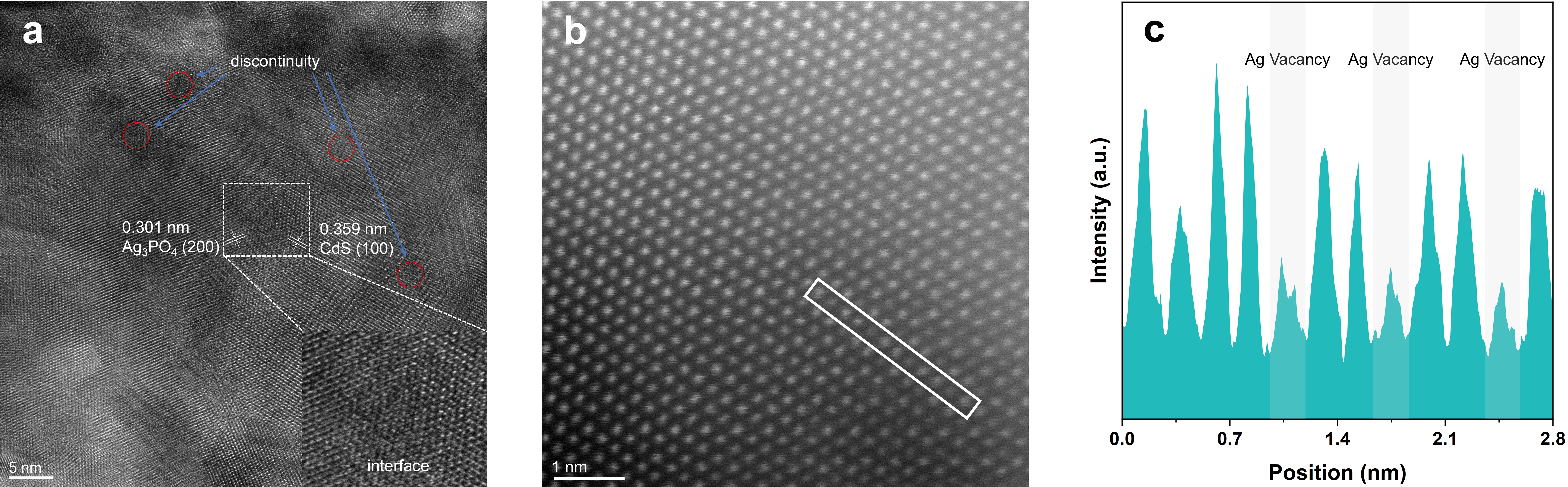


**Fig. S8** (**a**) HR-TEM image of Ag_3_PO_4_/CdS chip. The lattice-fringe spacing of 0.359 and 0.301 nm can be observed, corresponding to the (100) crystallographic planes of CdS and (200) crystallographic planes of Ag_3_PO_4_, respectively. The discontinuity is also visible, which provides visual evidence for the existence of S and Ag vacancies. AC-TEM image of Ag_3_PO_4_/CdS chip (**b**) and corresponding intensity profile (**c**). Dark spots at Ag sites are discernible. To quantify the defective sites’ brightness, we plot the integrated pixel intensities of the line extracted along the vacancy-involved atomic column marked by the white rectangle in (**b**)


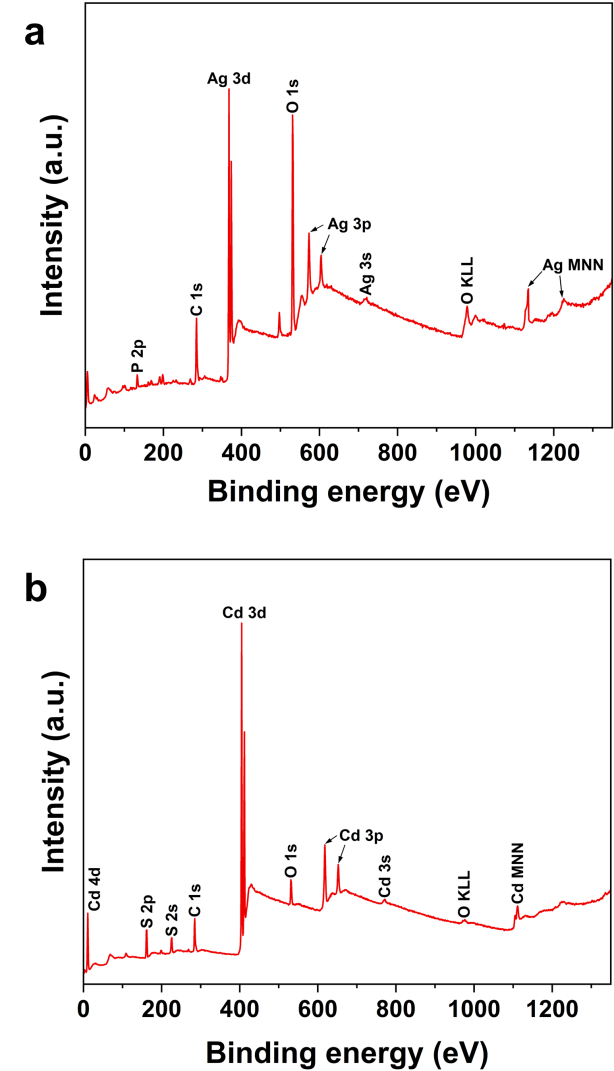

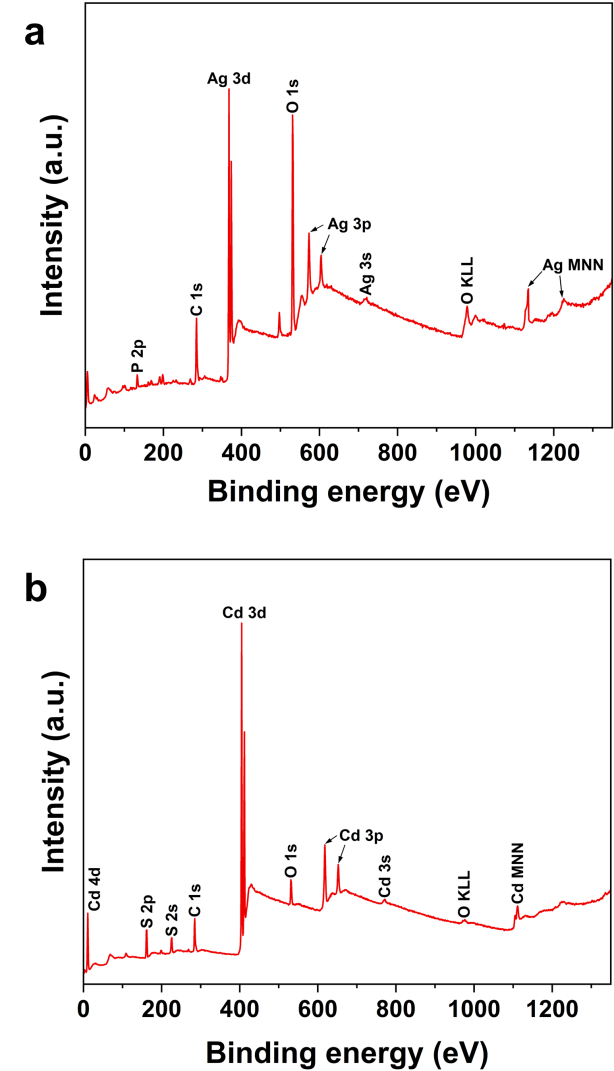


**Fig. S9** XPS spectra of Ag_3_PO_4_ and CdS thin films. (**a**) XPS spectrum of Ag_3_PO_4_ thin film. All elements of Ag, P, and O can be observed in the XPS survey spectrum. (**b**) XPS spectrum of CdS thin film. All elements of Cd and S can be observed in the XPS survey spectrum. The binding energy was calibrated by at 284.8 eV with respect to the carbon (C 1s) as a reference line


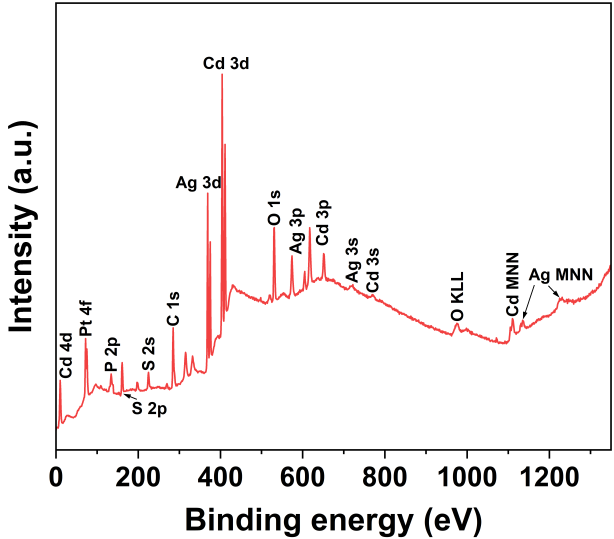


**Fig. S10** XPS spectrum of Ag_3_PO_4_/CdS porous microreactor chip photocatalyst. All elements of Ag, P, O, Cd, S and Pt can be found in the XPS survey spectrum and the binding energy was calibrated by at 284.8 eV with respect to the carbon (C 1s) as a reference line


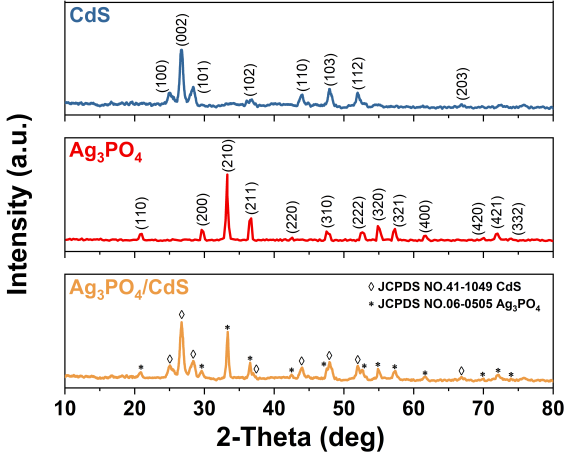


**Fig. S11** XRD pattern of Ag_3_PO_4_ layer, CdS layer and Ag_3_PO_4_/CdS interface


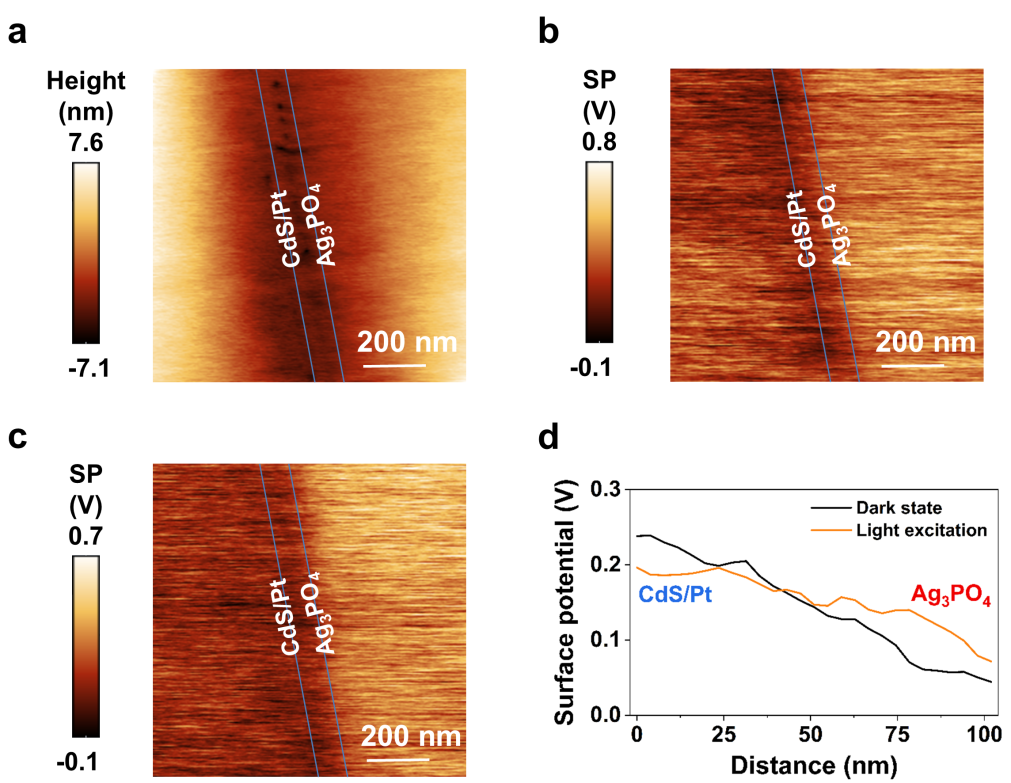


**Fig. S12** (**a**) AFM topography of the thinner Ag_3_PO_4_/CdS/Pt heterojunction. Surface potential images of the thinner Ag_3_PO_4_/CdS/Pt heterojunction in the dark (**b**) and under light (**c**). (**d**) Surface potential profiles at the interface of CdS/Pt and Ag_3_PO_4_ layers in the dark and under light


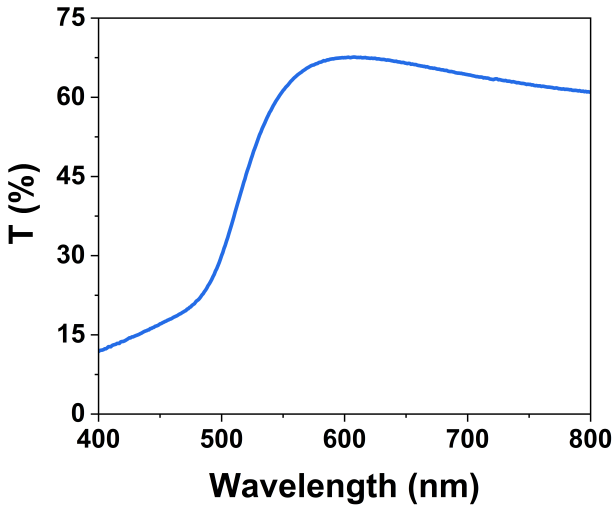


**Fig. S13** Visible transmittance spectrum of CdS/Pt thin film. Using the preparation process shown in Fig. 1a, 60-nm-thick CdS thin film and 0.3-nm Pt are successively deposited on a piece of 20 mm × 20 mm K9 glass, and then the visible transmittance spectrum of CdS/Pt thin film is obtained by a UV-visible-NIR spectrophotometer


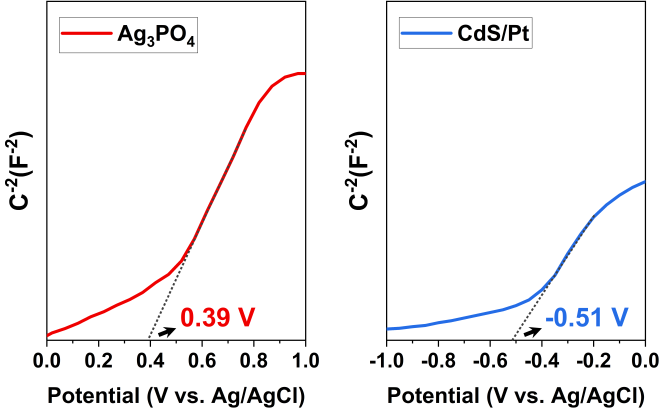


**Fig. S14** Mott-Schottky plots of Ag_3_PO_4_ and CdS/Pt. The flat band potentials of Ag_3_PO_4_ and CdS/Pt are 0.39 and -0.51 V versus Ag/AgCl, so the CB positions of Ag_3_PO_4_ and CdS/Pt are determined as 0.19 and -0.71 V versus Ag/AgCl, corresponding to 0.39 and -0.51 V (vs. NHE, pH = 0)


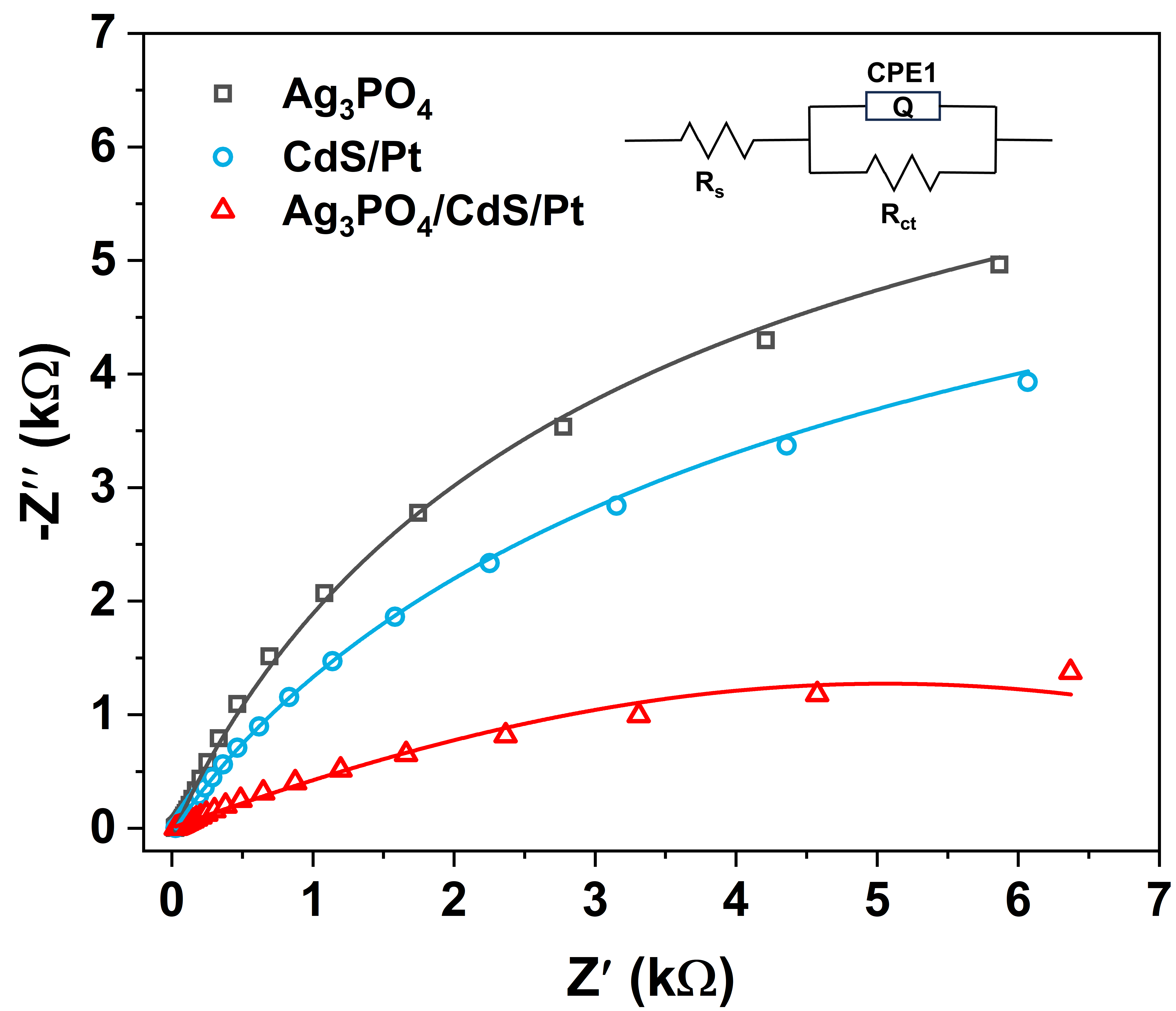


**Fig. S15** EIS Nyquist plots of as-prepared samples. The fitting method has been used in the literatures [S7, S8]. The arc radius of EIS Nyquist plots of Ag_3_PO_4_/CdS/Pt heterojunction is much smaller than that of Ag_3_PO_4_ and CdS/Pt, indicating that the electron transfer ability of the Ag_3_PO_4_/CdS/Pt heterojunction is much better than that of Ag_3_PO_4_ and CdS/Pt


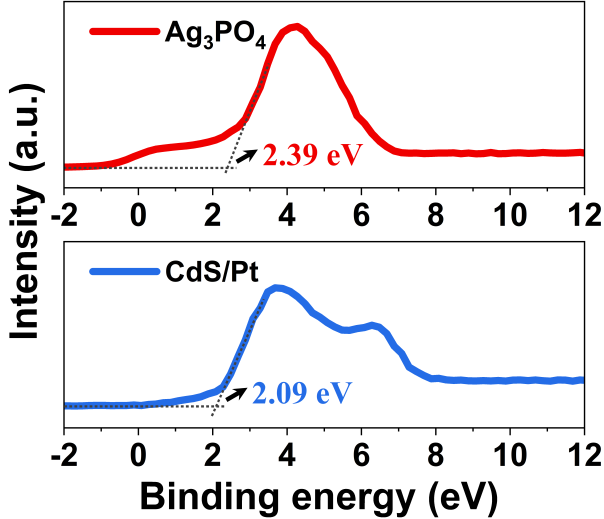


**Fig. S16** XPS VB spectra of Ag_3_PO_4_ and CdS/Pt layers of Ag_3_PO_4_/CdS/Pt heterojunction





**Fig. S17** (**a**) Band structure diagram for Ag_3_PO_4_ and CdS. Band structure diagram for Ag_3_PO_4_/CdS heterojunction with thickness greater than the space charge region in equilibrium (**b**) and under light (**c**). As for Ag_3_PO_4_/CdS heterojunction with thickness greater than the space charge region, the Fermi level (FL) difference between Ag_3_PO_4_ and CdS induces an upward band bending in CdS and a downward band bending in Ag_3_PO_4_, which weakens under illumination due to the photogenerated charge transferring to the surface. (**d**) Band structure diagram for Ag_3_PO_4_ and CdS. Band structure diagram for Ag_3_PO_4_/CdS heterojunction with thickness smaller than the space charge region in equilibrium (**e**) and under light (**f**). Tuning the thickness of Ag_3_PO_4_/CdS heterojunction within the space charge region will effectively avoid the band bending, and the band bending also weakens under illumination


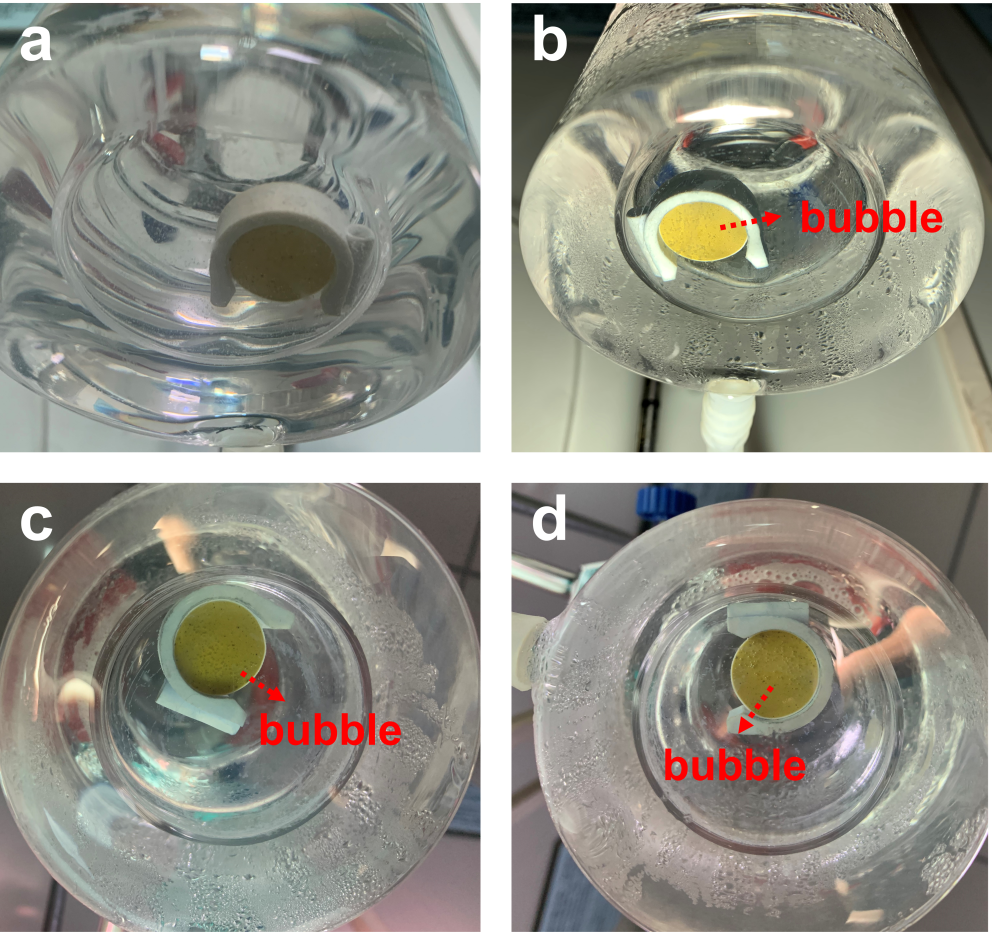


**Fig. S18** Optical images of seawater-splitting system operating at atmospheric pressure and room temperature before the start of the experiment (**a**) and at various time points, for example, the first hour (**b**), the third hour (**c**) and the seventh hour of the test (**d**). Before the experiment, there were no bubbles on the sample surface. After the beginning of the experiment, the samples were photographed at different time points and it was found that the dense bubbles appeared on the surface of the sample caused by the gas evolution


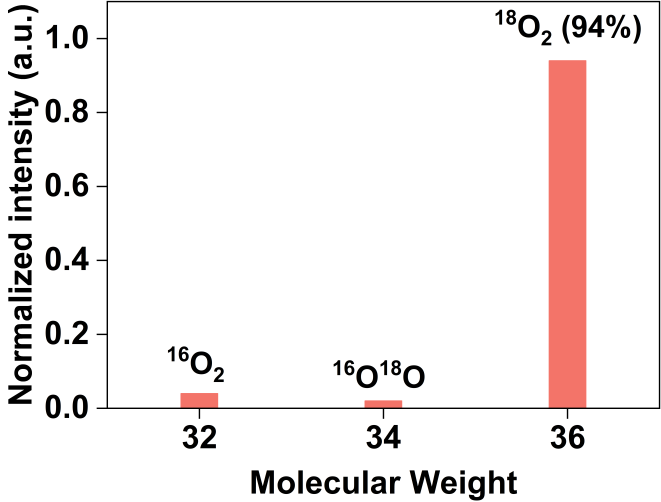


**Fig. S19** The mass spectrum of evolved O_2_ during photocatalytic 3.5 wt% NaCl solution made of H_2_^18^O splitting





**Fig. S20** Typical time course of H_2_ and O_2_ production. Photocatalytic activity of the Ag_3_PO_4_/CdS porous microreactor chip photocatalysts operating in pure water (**a**) and artificial seawater (**b**)


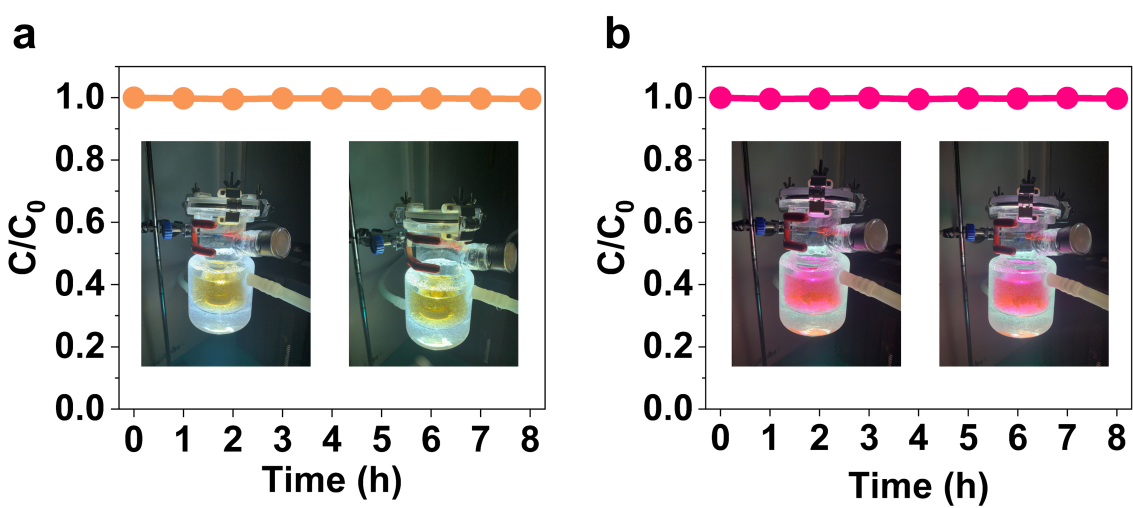


**Fig. S21** Reaction performance of the as-prepared Ag_3_PO_4_/CdS porous microreactor chip photocatalysts for photocatalytic degradation of MO (**a**) and RhB (**b**). Insets are the optical images of reaction system in the reaction stage. A negligible decrease in MO and RhB concentration and no color change were observed throughout the experiment, implying that the existence of vacancies on the sample surface made the dyes not involved in the competitive reaction for hydrogen and oxygen evolution


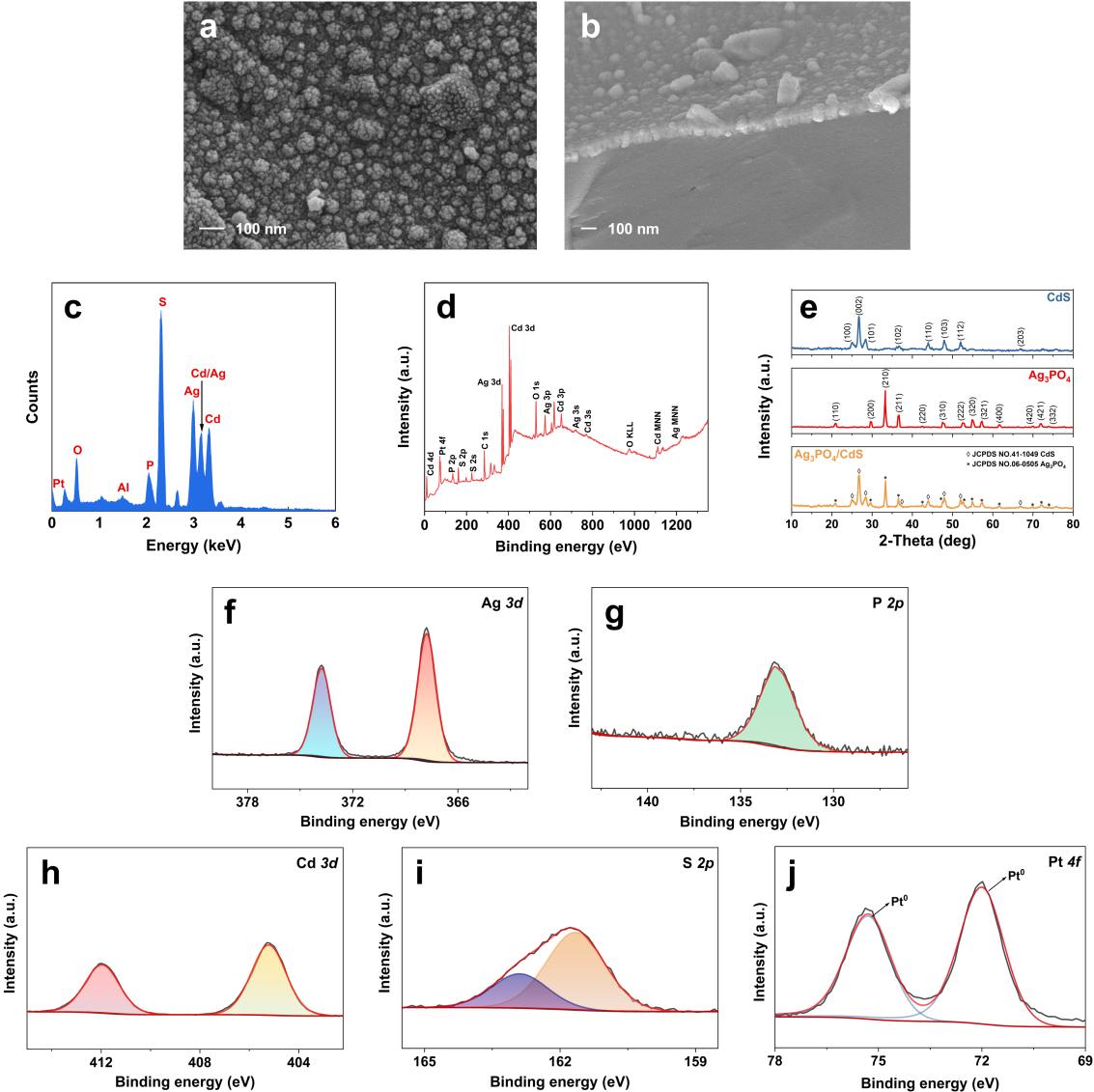


**Fig. S22** SEM image (top view) (**a**), SEM image (cross section) (**b**), EDS spectrum (**c**), XPS survey spectra (**d**), XRD pattern (**e**) and high-resolution XPS spectra (**f**–**j**) of Ag_3_PO_4_/CdS porous microreactor chip photocatalyst after photocatalytic overall seawater splitting reactions. After 300-h seawater splitting reaction, the morphology, chemical composition, elemental chemical state and crystal form of sample remain almost unchanged as compared to those before photocatalytic reaction


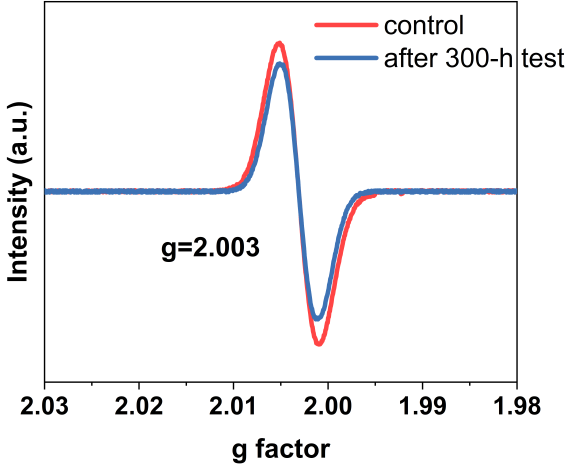


**Fig. S23** EPR pattern of Ag_3_PO_4_/CdS porous microreactor chip photocatalyst after photocatalytic overall seawater splitting reactions. After 300-h seawater splitting reaction, the EPR signal indicating the presence of S vacancy decreased by 14%


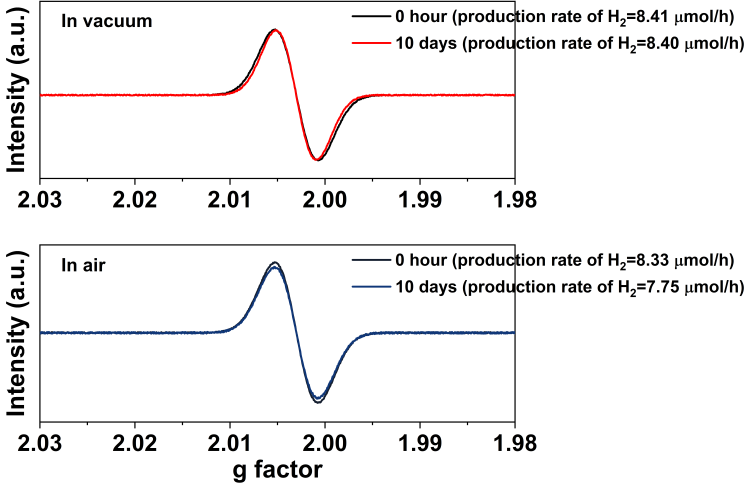


**Fig. S24** EPR patterns and corresponding activity data of the Ag_3_PO_4_/CdS chips under different storage conditions. After being stored in vacuum for 10 days, the sulfur vacancy concentration of the samples hardly changed, and the activity also remained unchanged. After 10 days of storage in air, the sulfur vacancy concentration and activity of another sample decreased by 6% and 7%, respectively


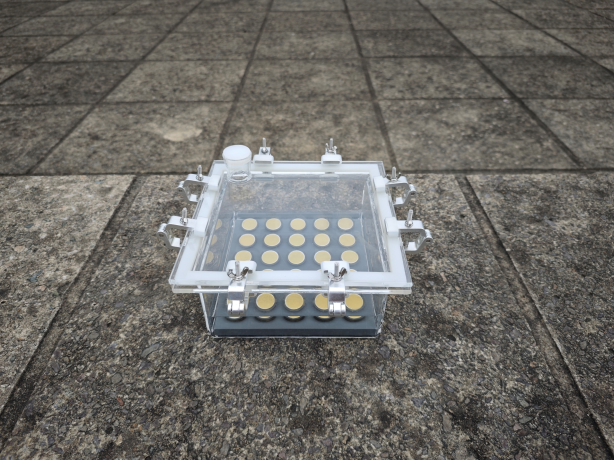


**Fig. S25** an optical image of outdoor set-up


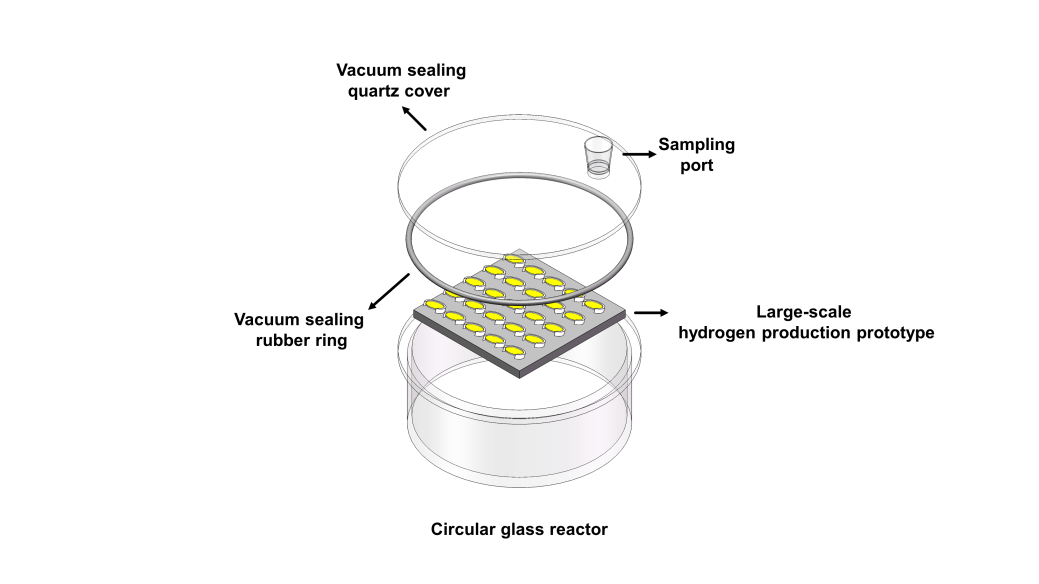


**Fig. S26** The design of a circular glass reactor. In some countries and regions, different architectural designs or terrains require different shapes of reactors

**Table S1** The geometric mean electronegativity of the semiconductors

|  | X_Cd_ | X_S_ | **X_CdS_** | X_Ag_ | X_P_ | X_O_ | **X_Ag3PO4_** |
| --- | --- | --- | --- | --- | --- | --- | --- |
| Value (eV) | 4.33 | 6.2 | **5.18** | 4.44 | 5.62 | 7.54 | **5.96** |

**Table S2** Comparison of AQY for photocatalytic water splitting over reported Ag_3_PO_4_-based and CdS-based photocatalysts

| **Catalysts** | **Sacrificial agent** | **AQY** | **Refs.** |
| --- | --- | --- | --- |
| Ag_3_PO_4_/CdS  porous microreactor chip | N/A | 12.26% at 420 nm | This work |
| CdS@NiO | N/A | 1.6% at 420 nm | [S9] |
| Pt-loaded TiO_2_/  CdS−(ZnSe)_0.5_(CuGa_2.5_Se_4.25_)_0.5_ | N/A | 1.5% at 420 nm | [S10] |
| Ag_3_PO_4_/SrTiO_3_ | AgNO_3_ | 16.2% at 420 nm | [S11] |
| Ag_3_PO_4_ | AgNO_3_ | ~80% at 420 nm | [S12] |
| TaON/CdS | lactic acid | 18.23% at 420 nm | [S13] |
| Ni-loaded CdS | (NH_4_)_2_SO_3_ | 48.2% at 420 nm | [S14] |
| MoS_2_/WS_2_/CdS | lactic acid | 58.94% at 420 nm | [S15] |
| NiS-loaded CdS | lactic acid | 65.7% at 420 nm | [S16] |

**Table S3** STH efficiency of Ag_3_PO_4_/CdS porous microreactor chip photocatalysts using pure water and artificial seawater as reaction solution

| **Reaction solution** | **H_2_ evolution rate (μmol h^-1^)** | **STH efficiency (%)** |
| --- | --- | --- |
| Pure water | 6.50 | 0.71 |
| Seawater | 8.37 | 0.92 |
| Artificial seawater | 15.70 | 1.72 |

**Table S4** The photoactivity of Ag_3_PO_4_/CdS porous microreactor chip photocatalysts in whole cycle testing

| **Days** | **H_2_ evolution rate (μmol h^-1^)** | **STH efficiency (%)** |
| --- | --- | --- |
| 1 | 8.37 | 0.92 |
| 2 | 8.19 | 0.90 |
| 3 | 7.92 | 0.87 |
| 4 | 7.79 | 0.85 |
| 5 | 7.68 | 0.84 |
| 6 | 7.63 | 0.84 |
| 7 | 7.92 | 0.87 |
| 8 | 7.83 | 0.86 |
| 9 | 7.64 | 0.84 |
| 10 | 7.43 | 0.81 |
| 11 | 7.28 | 0.80 |
| 12 | 7.08 | 0.78 |
| 13 | 7.71 | 0.85 |
| 14 | 7.47 | 0.82 |
| 15 | 7.32 | 0.80 |
| 16 | 7.08 | 0.78 |
| 17 | 7.06 | 0.77 |
| 18 | 6.74 | 0.74 |
| 19 | 7.39 | 0.81 |
| 20 | 7.14 | 0.78 |
| 21 | 6.95 | 0.76 |
| 22 | 6.72 | 0.74 |
| 23 | 6.72 | 0.74 |
| 24 | 6.51 | 0.71 |
| 25 | 7.04 | 0.77 |

**Table S5.** Comparison of several studies about photocatalytic overall seawater splitting

| **Catalysts** | **Reaction medium** | **Reaction conditions** | **Durability** | **Refs.** |
| --- | --- | --- | --- | --- |
| Ag_3_PO_4_/CdS porous microreactor chip | Natural seawater | Atmospheric pressure and room temperature | 300h | This work |
| NiO/Ni/La_2_Ti_2_O_7_ | Natural seawater with pretreatment | N/A | 3h | [S17] |
| (Ga_1-x_Zn_x_)(N_1-x_O_x_) | Artificial seawater | Room temperature | 5h | [S18] |
| p-GaN/InGaN nanowire array |  | N/A | 3h | [S19] |
| Pt/GaP-TiO_2_-SiO_2_:Rh |  | Room temperature | 12h | [S20] |
| InGaN/GaN nanowire array |  | High temperature (70 °C) | 10h | [S21] |

**Supplementary References**

1. S. A. Mirsalari, A. Nezamzadeh-Ejhieh, Focus on the photocatalytic pathway of the CdS-AgBr nano-catalyst by using the scavenging agents. Sep. Purif. Technol. **250**, 117235 (2020). <https://doi.org/10.1016/j.seppur.2020.117235>
2. N. Raeisi-Kheirabadi, A. Nezamzadeh-Ejhieh, A Z-scheme g-C_3_N_4_/Ag_3_PO_4_ nanocomposite: Its photocatalytic activity and capability for water splitting. Int. J. Hydrogen Energy **45**, 33381−33395 (2020). <https://doi.org/10.1016/j.ijhydene.2020.09.028>
3. Z. Wang, C. Li, K. Domen, Recent developments in heterogeneous photocatalysts for solar-driven overall water splitting. Chem. Soc. Rev. **48**, 2109−2125 (2019). <https://doi.org/10.1039/c8cs00542g>
4. A. Kudo, Y. Miseki, Heterogeneous photocatalyst materials for water splitting. Chem. Soc. Rev. **38**, 253−278 (2009). <https://doi.org/10.1039/b800489g>
5. D. Zhao, Y. Wang, C.-L. Dong, Y.-C. Huang, J. Chen et al., Boron-doped nitrogen-deficient carbon nitride-based Z-scheme heterostructures for photocatalytic overall water splitting. Nat. Energy **6**, 388–397 (2021). <https://doi.org/10.1038/s41586-021-03907-3>
6. Q. Wang, T. Hisatomi, Q. Jia, H. Tokudome, M. Zhong et al., Scalable water splitting on particulate photocatalyst sheets with a solar-to-hydrogen energy conversion efficiency exceeding 1%. Nat. Mater. **15**, 611–615 (2016). <https://doi.org/10.1038/NMAT4589>
7. Q. Zhao, B. Zhao, X. Long, R. Feng, M. Shakouri et al., Interfacial electronic modulation of dual-monodispersed Pt-Ni_3_S_2_ as efficacious bi-functional electrocatalysts for concurrent H_2_ evolution and methanol selective oxidation. Nano-Micro Lett. **16**, 80 (2024). [https://doi.org/ 10.1007/s40820-023-01282-4](https://doi.org/10.1038/s41467-022-28995-1)
8. X. Shi, C. Dai, X. Wang, J. Hu, J. Zhang et al., Protruding Pt single-sites on hexagonal ZnIn_2_S_4_ to accelerate photocatalytic hydrogen evolution. Nat. Commun. **13**, 1287 (2022). <https://doi.org/10.1038/s41467-022-28995-1>
9. S. Qiao, C. Feng, T. Chen, Y. Kou, W. Wang et al., Spherical shell CdS@NiO Z-scheme composites for solar-driven overall water splitting and carbon dioxide reduction. Mater. Today Energy **27**, 101044 (2022). <https://doi.org/10.1016/j.mtener.2022.101044>
10. S. Chen, J. J. M. Vequizo, Z. Pan, T. Hisatomi, M. Nakabayashi et al., Surface modifications of (ZnSe)_0.5_(CuGa_2.5_Se_4.25_)_0.5_ to promote photocatalytic Z-scheme overall water splitting. J. Am. Chem. Soc. **143**, 10633–10641 (2021). <https://doi.org/10.1021/jacs.1c03555>
11. X. Guan, L. Guo, Cocatalytic effect of SrTiO_3_ on Ag_3_PO_4_ toward enhanced photocatalytic water oxidation. ACS Catal. **4**, 3020−3026 (2018). [https://doi.org/ 10.1021/cs5005079](https://doi.org/10.1021/acscatal.8b03737)
12. Z. Yi, J. Ye, N. Kikugawa, T. Kako, S. Ouyang et al., An orthophosphate semiconductor with photooxidation properties under visible-light irradiation. Nat. Mater. **9**, 559–564 (2010). <https://doi.org/10.1038/nmat2780>
13. F. Chen, H. Fu, X. Yang, S. Xiong, X. An. Fabrication of TaON/CdS heterostructures for enhanced photocatalytic hydrogen evolution under visible light irradiation. Catalysts. **12**, 1110 (2022). <https://doi.org/10.3390/catal12101110>
14. Q. Zhao, J. Sun, S. Li, C. Huang, W. Yao et al., Single nickel atoms anchored on nitrogen-doped graphene as a highly active cocatalyst for photocatalytic H_2_ evolution. ACS Catal. **8**, 11863−11874 (2018). <https://doi.org/10.1021/acscatal.8b03737>
15. Y. Sun, B. Wang, X. Liu, L. Gao, W. Shangguan, Synthesis of ternary cross-linked MoS_2_/WS_2_/CdS photocatalysts for photocatalytic H_2_ production. Catalysts. **13**, 1149 (2023). <https://doi.org/10.3390/catal13081149>
16. X. Fan, B. Wang, Q. Heng, W. Chen, L. Mao, Facile in-situ synthesis of α-NiS/CdS p-n junction with enhanced photocatalytic H_2_ production activity. Int. J. Hydrogen Energy **47**, 32531−32542 (2022). <https://doi.org/10.1016/j.ijhydene.2022.07.265>
17. S. M. Ji, H. Jun, J. S. Jang, H. C. Son, P. H. Borse et al., Photocatalytic hydrogen production from natural seawater. J. Photochem. Photobiol. Chem. **189**, 141−144 (2007). <https://doi.org/10.1016/j.jphotochem.2007.01.011>
18. K. Maeda, H. Masuda, K. Domen, Effect of electrolyte addition on activity of (Ga_1-x_Zn_x_)(N_1-x_O_x_) photocatalyst for overall water splitting under visible light. Catal. Today, **147**, 173−178 (2009). <https://doi.org/10.1016/j.cattod.2008.09.002>
19. X. Guan, F. A. Chowdhury, N. Pant, L. Guo, L. Vayssieres et al., Efficient unassisted overall photocatalytic seawater splitting on GaN-based nanowire arrays. J. Phys. Chem. C. **122**, 13797–13802 (2018). <https://doi.org/10.1021/acs.jpcc.8b00875>
20. H. V. Dang, Y. H. Wang, J. C. S. Wu, Z-scheme photocatalyst Pt/GaP-TiO_2_-SiO_2_:Rh for the separated H_2_ evolution from photocatalytic seawater splitting. Appl. Catal. B-Environ. **296**, 120339 (2021). <https://doi.org/10.1016/j.apcatb.2021.120339>
21. P. Zhou, I. A. Navid, Y. Ma, Y. Xiao, P. Wang et al., Solar-to-hydrogen efficiency of more than 9% in photocatalytic water splitting. Nature **613**, 66–70 (2023). <https://doi.org/10.1038/s41586-022-05399-1>
